# Efficient permutation-based genome-wide association studies for normal and skewed phenotypic distributions

**DOI:** 10.1093/bioinformatics/btac455

**Published:** 2022-09-18

**Authors:** Maura John, Markus J Ankenbrand, Carolin Artmann, Jan A Freudenthal, Arthur Korte, Dominik G Grimm

**Affiliations:** Technical University of Munich, Campus Straubing for Biotechnology and Sustainability, Bioinformatics, 94315 Straubing, Germany; Weihenstephan-Triesdorf University of Applied Sciences, Bioinformatics, 94315 Straubing, Germany; Center for Computational and Theoretical Biology, University of Würzburg, 97078 Würzburg, Germany; Center for Computational and Theoretical Biology, University of Würzburg, 97078 Würzburg, Germany; Center for Computational and Theoretical Biology, University of Würzburg, 97078 Würzburg, Germany; Center for Computational and Theoretical Biology, University of Würzburg, 97078 Würzburg, Germany; Technical University of Munich, Campus Straubing for Biotechnology and Sustainability, Bioinformatics, 94315 Straubing, Germany; Weihenstephan-Triesdorf University of Applied Sciences, Bioinformatics, 94315 Straubing, Germany; Department of Informatics, Technical University of Munich, 85748 Garching, Germany

## Abstract

**Motivation:**

Genome-wide association studies (GWAS) are an integral tool for studying the architecture of complex genotype and phenotype relationships. Linear mixed models (LMMs) are commonly used to detect associations between genetic markers and a trait of interest, while at the same time allowing to account for population structure and cryptic relatedness. Assumptions of LMMs include a normal distribution of the residuals and that the genetic markers are independent and identically distributed—both assumptions are often violated in real data. Permutation-based methods can help to overcome some of these limitations and provide more realistic thresholds for the discovery of true associations. Still, in practice, they are rarely implemented due to the high computational complexity.

**Results:**

We propose permGWAS, an efficient LMM reformulation based on 4D tensors that can provide permutation-based significance thresholds. We show that our method outperforms current state-of-the-art LMMs with respect to runtime and that permutation-based thresholds have lower false discovery rates for skewed phenotypes compared to the commonly used Bonferroni threshold. Furthermore, using permGWAS we re-analyzed more than 500 *Arabidopsis thaliana* phenotypes with 100 permutations each in less than 8 days on a single GPU. Our re-analyses suggest that applying a permutation-based threshold can improve and refine the interpretation of GWAS results.

**Availability and implementation:**

permGWAS is open-source and publicly available on GitHub for download: https://github.com/grimmlab/permGWAS.

**Supplementary information:**

Supplementary data are available at *Bioinformatics* online.

## 1 Introduction

Genome-wide association studies (GWAS) are an integral tool to detect associations between genetic markers within a population of individuals and a complex trait or disease that is measured in the same population (Atwell *et al.*, 2010; The 1001 Genomes Consortium, 2016; Todesco *et al.*, 2020). State-of-the-art methods can easily analyze millions of markers in populations of thousands of individuals (Kang *et al.*, 2008, 2010; Korte *et al.*, 2012; Lippert *et al.*, 2011; Loh *et al.*, 2015). Here, the critical step is to define a threshold to distinguish true and spurious associations. Classically, one controls the family-wise error rate (FWER), that is the probability of making at least one type-1 error (or false positive), using the commonly used Bonferroni correction (Bonferroni, 1936). However, due to the large number of tests the Bonferroni correction is in practice often too conservative (Gumpinger *et al.*, 2021; Llinares-López *et al.*, 2015; Westfall and Young, 1993), as it assumes that all tested markers are independent, which is clearly not the case for high-density genomic data that are nowadays routinely generated. Here, many markers are correlated with each other and the actual number of independent tests performed is lower than the number of markers analyzed. Therefore, many studies propose a significance threshold that is based on the false discovery rate (FDR) (Storey and Tibshirani, 2003). On the other hand, naïve thresholds, such as Bonferroni or FDR, cannot account for model mis-specifications that easily arise in biological data, which are often non-normally distributed. Variance-stabilizing transformations have been proposed to account for phenotypic variability (Sun *et al.*, 2013), but are not non-controversial (Shen and Rönnegård, 2013) and might complicate comparability across different phenotypes. Permutation-based thresholds could provide an alternative approach to overcome some of these limitations (Che *et al.*, 2014). Here, the main limitations are the computational burden to run permutations routinely, as current implementations are still too slow and inefficient [such as our deprecated method GWAS-Flow (Freudenthal *et al.*, 2019)], or focus only on linear regression without the possibility to correct for confounding factors on specialized Field Programmable Gate Arrays hardware (Swiel *et al.*, 2022).

We propose permGWAS, an efficient permutation-based linear mixed model (LMM) to compute adjusted significance thresholds that are able to account for correlated markers and skewed phenotypic distributions without the need to arbitrarily transform phenotypes. To account for multiple hypotheses, correlated markers and skewed phenotypes, we compute permutation-based significance thresholds based on the *maxT* method proposed by Westfall and Young (1993). To enable efficient computation of different permutation-based tests, we provide a scaleable batch-wise reformulation of a permutation-based LMM using 4D tensors. We propose to implement permutation-based thresholds as the default choice for GWAS and provide both simulations and re-analysis of more than 500 *Arabidopsis thaliana* phenotypes to underpin its benefits.

## 2 Materials and methods

We will first provide the necessary background of LMMs for GWAS, the multiple hypothesis testing problem and how to empirically estimate the FWER using the Westfall–Young permutation testing procedure (Westfall and Young, 1993). Finally, we will present our approach on how to efficiently compute associations with LMMs using a permutation-based significance threshold. An overview of all mathematical symbols and notations can be found in Supplementary Table S1.

### 2.1 Linear mixed model

Let *n* be the number of samples and *m* the number of genetic markers. Then for each genetic marker we consider a LMM of the form

(1) y=Xβ+u+ϵ,

where $y\in {\mathbb{R}}^{n}$ is a vector of observed phenotypic values and $X\in {\mathbb{R}}^{n\times c}$ is a matrix of fixed effects containing columns for the mean, covariates and the genetic marker. Fixed effects are denoted by $\beta \in {\mathbb{R}}^{c}$ and random effects $u\in {\mathbb{R}}^{n}$ follow a Gaussian distribution with zero mean and a (genetic) variance of $\sigma_{g}^{2}K$, where $K\in {\mathbb{R}}^{n\times n}$ denotes the kinship matrix and $\epsilon \in {\mathbb{R}}^{n}$ is a vector of residual effects with $\epsilon ∼N(0,\sigma_{e}^{2}I)$. As described in Kang *et al.* (2008, 2010) and Lippert *et al.* (2011), we estimate the variance components $\sigma_{g}^{2}$ and $\sigma_{e}^{2}$ by maximizing the following likelihood function

(2) $$L\left(\beta ,\sigma_{g}^{2},\sigma_{e}^{2}\right)=N\left(y|X\beta ;\sigma_{g}^{2}K+\sigma_{e}^{2}I\right)$$

once for a null model, which includes no genetic markers and reuse them for the alternative model, which includes the marker of interest. Finally, an F-test is used to test the null hypothesis that the marker has no effect against the alternative hypothesis that it has an effect on the phenotypic value. We reject the null hypothesis and call a statistical test significant, if the *P*-value of the F-test is below a predefined significance threshold *α* (e.g. 5%).

### 2.2 Multiple hypothesis testing

Since we have to test thousands to millions of markers simultaneously, we have to take these multiple tests into account, otherwise we would obtain thousands of false positive associations deemed to be significant.

#### 2.2.1 Family-wise error rate

The FWER is the probability of making at least one type-1 error (or false positive). One has to find an appropriate corrected significance threshold *δ* for each hypothesis, such that the FWER(*δ*) ≤α. To determine the optimal threshold $\delta^{*}$ one has to solve the following optimization problem:

(3) $$\delta^{*}=\max\left\{\delta |\text{FWER}(\delta )\leq \alpha \right\}.$$

Evaluating this optimization problem in closed form is not possible in general. For this purpose, the widely used Bonferroni approximation (Bonferroni, 1936) can be used to control the FWER. To estimate the adjusted significance threshold $\delta_{b}^{*}$ after Bonferroni, one simply divides the target significance level *α* by the number of simultaneous tests, i.e. $\delta_{b}^{*}=\alpha /m$. However, due to the large number of tests, the Bonferroni correction is in practice often too conservative, i.e. FWER($\delta_{b}^{*}$) $\ll \delta^{*}$, as shown in Llinares-López *et al.* (2015) and Gumpinger *et al.* (2021). In addition, when performing GWAS one typically makes the assumption that the residuals are normally distributed and that the genetic markers are independent and identically distributed. However, these assumptions are often violated in practice, which leads to the fact that the Bonferroni threshold is either overly conservative for normally distributed phenotypes (leading to many false negatives) or not stringent enough for phenotypes with skewed distributions (leading to many false positives).

#### 2.2.2 Westfall–Young permutations

Permutation-based methods can help to overcome some of these problems, by empirically estimating the FWER(*δ*). One could either approximate the null distribution by using permutations to then compute adjusted *P*-values or use the unadjusted *P*-values and provide a permutation-based significance threshold based on the *maxT* permutation-method proposed by Westfall and Young (1993). With this adjusted threshold we can account for non-Gaussian distributed phenotypes, correlated markers due to linkage disequilibrium and the large number of tests. In the following we will describe how to compute both, adjusted *P*-values and adjusted significance thresholds.

To compute adjusted *P*-values, we first permute the phenotype *q* times and calculate the test statistic ${}^{\left(k\right)}t_{j}$ for the *k*th permutation, with k∈{1,…,q} and *j*th marker, with j∈{1,…,m}. After randomizing, any correlation left between the genotypic and phenotypic values will be of non-genetic origin, but the distribution of the phenotypic values stays the same. To compute the permutation-based *P*-values, let *T_j_* denote the random variable corresponding to the observed test statistic of the *j*th marker. We test the hypothesis *H*_0_ that *T_j_* follows the permutation distribution empirically given by ${}^{\left(k\right)}t_{j}$ for all *k* and all *j*. Then we compute the adjusted permutation-based *P*-value as:

(4) $$p_{j}=P\left(T_{j}=t_{j}|H_{0}\right)=\frac{\sum_{j=1}^{m}\sum_{k=1}^{q}1\left({}^{\left(k\right)}t_{j}\geq t_{j}\right)}{qm},$$

where 1 takes the value 1 if the argument is true and 0 otherwise. The FWER can be controlled in this multiple hypothesis testing setting using Bonferroni (1936).

For the adjusted significance threshold we follow a permutation testing procedure proposed by Westfall and Young (1993). For each permutation we take the maximal test statistic over all markers, ${}^{\left(k\right)}t_{\text{max}}=\max_{j\in \{1,\ldots ,m\}}{}^{\left(k\right)}t_{j}$ and compute the corresponding minimal *P*-value ${}^{\left(k\right)}p_{\text{min}}$. Let again *T_j_* denote the random variable corresponding to the observed test statistic of the *j*th marker. We now test the hypothesis *H*_0_ that *T_j_* follows the permutation distribution empirically given by ${}^{\left(k\right)}t_{\text{max}}$ for all *k*. Then the adjusted *P*-value is given by ${\tilde{p}}_{j}=P\left(T_{j}=t_{j}|H_{0}\right)$ and

(5) $${\tilde{p}}_{j}=\frac{\sum_{k=1}^{q}1\left({}^{\left(k\right)}t_{\text{max}}\geq t_{j}\right)}{q}\leq \alpha$$

is equivalent to *t_j_* being larger than the 100(1−α)th percentile of the ${}^{\left(k\right)}t_{\text{max}}$. Hence, the αth percentile of the minimal *P*-values ${}^{\left(k\right)}p_{\text{min}}$ leaves us with an adjusted threshold that controls the FWER.

### 2.3 permGWAS architecture

These permutation-based strategies are computationally highly demanding, which makes them often inapplicable in practice. Further, current state-of-the-art GWAS implementations sequentially compute univariate test statistics for one marker and a given phenotype (Grimm *et al.*, 2017; Kang *et al.*, 2008, 2010; Lippert *et al.*, 2011). We propose permGWAS, which is able to simultaneously compute univariate test statistics of several SNPs batch-wise on modern multi-CPU and GPU environments, while at the same time controlling the FWER using Westfall–Young permutation testing. First, we will introduce the mathematical framework for batch-wise LMM without permutations, followed by an efficient formulation for permutation-based LMM.

#### 2.3.1 Batch-wise linear mixed models

**Fig. 1.**
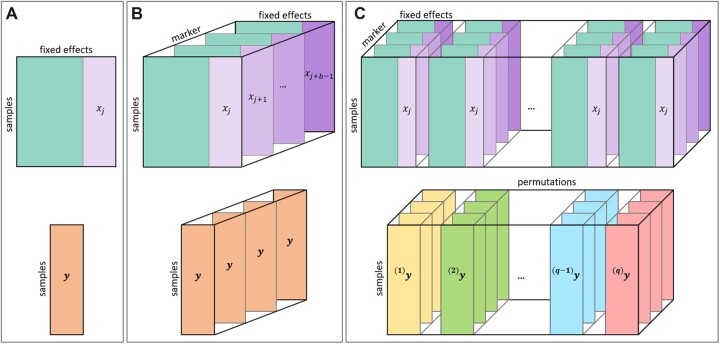
Schematic illustration of matrices and tensors of the permGWAS architecture. (**A**) Commonly used matrix representation when computing sequential univariate tests, where $y\in {\mathbb{R}}^{n}$ is the phenotypic vector for *n* samples and $X_{j}\in {\mathbb{R}}^{n\times c}$ denotes the matrix of fixed effects, including a column of ones for the intercept, the covariates and the *j*th SNP $x_{j}\in {\mathbb{R}}^{n}$. (**B**) 3D-tensor representation of a LMM to compute univariate tests batch-wise. The phenotype is represented as a 3D tensor containing *b* copies of the phenotype vector $y\in {\mathbb{R}}^{n}$ and $X_{j}^{b}\in {\mathbb{R}}^{b\times n\times c}$ is a 3D tensor containing the matrices $X_{j}$ to $X_{j+b-1}$. (**C**) 4D-tensor representation of a permutation-based batch-wise LMM. The phenotype is represented as a 4D tensor containing for each permutation ${}^{\left(k\right)}y$ the 3D tensor ${}^{\left(k\right)}Y^{b}$ for all *q* permutations and ${}^{q}X_{j}^{b}\in {\mathbb{R}}^{q\times b\times n\times c}$ is a 4D tensor containing *q* copies of $X_{j}^{b}$

Denote by *n* the number of samples, *c* the number of fixed effects (i.e. the SNP of interest and all covariates) and *b* the batch size. Let $X_{j}\in {\mathbb{R}}^{n\times c}$ denote the matrix of fixed effects, including a column of ones for the intercept, the covariates and the jth SNP $x_{j}\in {\mathbb{R}}^{n}$ (Fig. 1A). Let $X_{j}^{b}\in {\mathbb{R}}^{b\times n\times c}$ be the 3D tensor containing the matrices $X_{j}$ to $X_{j+b-1}$ and let $Y^{b}\in {\mathbb{R}}^{b\times n\times 1}$ denote the 3D tensor containing *b* copies of the phenotype vector $y\in {\mathbb{R}}^{n}$ (Fig. 1B). Further, let $V=\sigma_{g}^{2}K+\sigma_{e}^{2}I\in {\mathbb{R}}^{n\times n}$ denote the variance–covariance matrix. For computational efficiency, instead of using generalized least squares, we first compute the Cholesky decomposition $V=CC^{T}$ and linearly transform $X_{j}^{b}$ and $Y^{b}$, before computing the coefficients using ordinary least squares. Let $C^{b}\in {\mathbb{R}}^{b\times n\times n}$ denote the 3D tensor containing *b* copies of ***C***. Then, the linearly transformed data is given by

(6) $${\tilde{X}}_{j}^{b}={\left(C^{b}\right)}^{-1}X_{j}^{b}=\left(C^{-1}X_{j},\ldots ,C^{-1}X_{j+b-1}\right)$$

 

(7) $${\tilde{Y}}^{b}={\left(C^{b}\right)}^{-1}Y^{b}=\left(C^{-1}y,\ldots ,C^{-1}y\right).$$

Now, we can compute the coefficients $\beta_{j}^{b}\in {\mathbb{R}}^{b\times c}$ and the residual sums of squares ${\mathrm{RSS}}_{j}^{b}\in {\mathbb{R}}^{b}$ for those *b* SNPs starting at the jth SNP as follows:

(8) $$\beta_{j}^{b}={\left({\left({\tilde{X}}_{j}^{b}\right)}^{T}{\tilde{X}}_{j}^{b}\right)}^{-1}{\left({\tilde{X}}_{j}^{b}\right)}^{T}{\tilde{Y}}^{b}$$

 

(9) $${\mathrm{RSS}}_{j}^{b}={\left({\tilde{Y}}^{b}-{\tilde{X}}_{j}^{b}\beta^{b}\right)}^{T}\left({\tilde{Y}}^{b}-{\tilde{X}}_{j}^{b}\beta^{b}\right)$$

where ${\left({\tilde{X}}_{j}^{b}\right)}^{T}=\left({\left(C^{-1}X_{j}\right)}^{T},\ldots ,{\left(C^{-1}X_{j+b-1}\right)}^{T}\right)\in {\mathbb{R}}^{b\times c\times n}$. Finally, we can compute the test statistics $t_{j}^{b}\in {\mathbb{R}}^{b}$ for all *b* SNPs:

(10) $$t_{j}^{b}=(n-c)\frac{{\mathrm{RSS}}_{0}^{b}-{\mathrm{RSS}}_{j}^{b}}{{\mathrm{RSS}}_{j}^{b}}$$

where ${\mathrm{RSS}}_{0}^{b}$contains *b* copies of the residual sum of squares of the null model. Once we have computed the test statistics for all SNPs, we can sequentially calculate all *P*-values.

#### 2.3.2 Efficient permutation-based linear mixed models

When performing GWAS with permutations, let additionally *q* denote the number of permutations. Then for each permutation ${}^{\left(k\right)}y$ of *y* with k∈{1,…,q}, we get a new 3D tensor ${}^{\left(k\right)}Y^{b}\in {\mathbb{R}}^{b\times n\times 1}$. Let ${}^{q}Y^{b}\in {\mathbb{R}}^{q\times b\times n\times 1}$ be the 4D tensor containing ${}^{\left(k\right)}Y^{b}$ for all *k* and let ${}^{q}X_{j}^{b}\in {\mathbb{R}}^{q\times b\times n\times c}$ be the 4D tensor containing *q* copies of $X_{j}^{b}$ (Fig. 1C). Now for each permutation ${}^{\left(k\right)}y$ of *y*, we estimate associated variance components ${}^{\left(k\right)}\sigma_{g}^{2}$ and ${}^{\left(k\right)}\sigma_{e}^{2}$ and obtain a new variance–covariance matrix ${}^{\left(k\right)}V\in {\mathbb{R}}^{n\times n}$. We compute the Cholesky decomposition ${}^{\left(k\right)}V{=}^{\left(k\right)}C^{\left(k\right)}C^{T}$ for each *k* and again linearly transform the data. Let ${}^{q}C^{b}\in {\mathbb{R}}^{q\times b\times n\times n}$ denote the 4D tensor containing the 3D tensors ${}^{\left(k\right)}C^{b}\in {\mathbb{R}}^{b\times n\times n}$ for all *k*. Then, we can transform the data via

(11) $${}^{q}{\tilde{X}}_{j}^{b}={\left({}^{q}C^{b}\right)}^{-1}{}^{q}X_{j}^{b}=\left({\left({}^{\left(1\right)}C^{b}\right)}^{-1}X_{j}^{b},\ldots ,{\left({}^{\left(q\right)}C^{b}\right)}^{-1}X_{j}^{b}\right)$$

 

(12) $${}^{q}{\tilde{Y}}^{b}={\left({}^{q}C^{b}\right)}^{-1}{}^{q}Y^{b}=\left({\left({}^{\left(1\right)}C^{b}\right)}^{-1}{}^{\left(1\right)}Y^{b},\ldots ,{\left({}^{\left(q\right)}C^{b}\right)}^{-1}{}^{\left(q\right)}Y^{b}\right)$$

Now similar to above, we compute the coefficients ${}^{q}\beta_{j}^{b}\in {\mathbb{R}}^{q\times b\times c}$, the residual sums of squares ${}^{q}{\mathrm{RSS}}_{j}^{b}\in {\mathbb{R}}^{q\times b}$ and the test statistics ${}^{q}t_{j}^{b}\in {\mathbb{R}}^{q\times b}$ for all *q* permutations and *b* SNPs at once:

(13) $${}^{q}\beta_{j}^{b}={\left({\left({}^{q}{\tilde{X}}_{j}^{b}\right)}^{T}{}^{q}{\tilde{X}}_{j}^{b}\right)}^{-1}{\left({}^{q}{\tilde{X}}_{j}^{b}\right)}^{T}{}^{q}{\tilde{Y}}^{b}$$

 

(14) $${}^{q}{\mathrm{RSS}}_{j}^{b}={\left({}^{q}{\tilde{Y}}^{b}{-}^{q}{\tilde{X}}_{j}^{b}{}^{q}\beta_{j}^{b}\right)}^{T}\left({}^{q}{\tilde{Y}}^{b}{-}^{q}{\tilde{X}}_{j}^{b}{}^{q}\beta_{j}^{b}\right)$$

 

(15) $${}^{q}t_{j}^{b}=(n-c)\frac{{}^{q}{\mathrm{RSS}}_{0}^{b}{-}^{q}{\mathrm{RSS}}_{j}^{b}}{{}^{q}{\mathrm{RSS}}_{j}^{b}}$$

where

(16) $${\left({}^{q}{\tilde{X}}_{j}^{b}\right)}^{T}=\left({\left({\left({}^{\left(1\right)}C^{b}\right)}^{-1}X_{j}^{b}\right)}^{T},\ldots ,{\left({\left({}^{\left(q\right)}C^{b}\right)}^{-1}X_{j}^{b}\right)}^{T}\right)$$

is a 4D tensor in ${\mathbb{R}}^{q\times b\times c\times n}$ and ${}^{q}{\mathrm{RSS}}_{0}^{b}\in {\mathbb{R}}^{q\times b}$ contains *b* copies of the RSS of the null model for each permutation.

### 2.4 Implementation

The permGWAS framework is implemented in Python3 using commonly used libraries for scientific computing, such as numpy (Harris *et al.*, 2020), scipy (Virtanen *et al.*, 2020), pandas (McKinney *et al.*, 2011) and PyTorch (Paszke *et al.*, 2019) to support efficient tensor arithmetic as well as multi-core and GPU support. In addition, specialized packages for estimating the variance components [limix (Lippert *et al.*, 2014)] and file IO (h5py, pandas-plink) are used. permGWAS can be used as a standalone command line tool or directly within Python. Our implementation allows both, the usage of multi-core CPU architectures with and without GPU support. To ensure a smooth experience on different environments and machines, we provide a standardized Docker environment. Our framework supports several common genotype and phenotype file formats, including HDF5, CSV and PLINK (Purcell *et al.*, 2007). Further, permGWAS supports filtering for minor allele frequency and also including one or more covariates to account for certain fixed effects. By default, permGWAS computes as a kinship matrix the realized relationship kernel (Hayes *et al.*, 2009); however, it is also possible to provide any other type of genetic similarity matrix. In order to run the tool on different machines, the batch size for the simultaneous computation of univariate tests as well as the batch size for permutation-based tests can be adjusted. To reduce the memory footprint, it is also possible to load genotypic data continuously in chunks from a HDF5 file, in case a pre-computed kinship matrix is provided. All code is open-source and publicly available on GitHub, including more details and information on how to run the tool: https://github.com/grimmlab/permGWAS.

### 2.5 Data and simulations

We evaluate the performance and runtime of permGWAS on simulated data as well as on publicly available genotype and phenotype data from the model plant *A.thaliana*.

#### 2.5.1 *Arabidopsis thaliana* data

As genotypic data a fully imputed SNP-Matrix of 2029 accessions and approximately 3M segregating markers is used (Arouisse *et al.*, 2020). Phenotypic data for 516 different traits were downloaded from the central and manually curated AraPheno database (Seren *et al.*, 2016; Togninalli *et al.*, 2020).

#### 2.5.2 Simulations

Artificial phenotypes were simulated for 200 random *A.thaliana* accessions using the fully imputed SNP matrix from above. For each synthetic phenotype, 1001 SNPs with a minor allele frequency of 5% or higher were randomly sampled, where 1 SNP was considered causative and the other 1000 were used to simulate the polygenic background. Here, each background SNP contributed a small random amount, drawn from a normal distribution with *μ *= 0 and σ=0.1 to the phenotypic value. Random noise drawn from a gamma or normal distribution was added, such that the polygenic background accounts for 70% of the total phenotypic variance. Finally, a fixed effect for the causative SNP was added to explain roughly 20% of the total genetic variance. In this manner, 6 different sets containing 50 phenotypes each, were simulated. The sets differed by the distribution of the noise, where 1 set had normally distributed noise and the other 5 sets used gamma-distributed noise with shape parameters of 0.1, 1, 2, 3 and 4. For evaluation, permGWAS was applied with 100 permutations on each of the 300 simulated phenotypes. Each phenotype was classified as true positive (TP) if any SNP in a 50 kbp window around the causative marker was significant. In addition, each phenotype was classified as false positive (FP) if any SNP outside the 50 kbp window around the causative marker was significant. In this way, a phenotype can be true positive and false positive at the same time. We define the phenotype-wise FDR as FDR := $\frac{\mathrm{FP}}{\mathrm{TP}+\mathrm{FP}\;}$. These values were calculated separately for the *P*-value thresholds based on both, the Bonferroni and permutation-based thresholds.

## 3 Results and discussion

In the following, we evaluate permGWAS with respect to runtime and statistical power using simulated data, as well as more than 500 public available phenotypes from the model species *A.thaliana*.

### 3.1 Results on synthetic data

#### 3.1.1 Runtime comparisons

We analyzed the runtime of permGWAS with respect to (i) the number of markers, (ii) the number of samples and (iii) the number of permutations. For all runtime experiments, we used data from a flowering time related phenotype in *A.thaliana*, FT10 (flowering time at 10 degrees; DOI: 10.21958/phenotype:261) (The 1001 Genomes Consortium, 2016), and down- and up-sampled the phenotype and corresponding SNP matrix to generate synthetic data with varying numbers of samples. We compared the runtime of permGWAS with two state-of-the-art and commonly used LMMs, EMMAX (Kang *et al.*, 2010) and FaST-LMM (Lippert *et al.*, 2011). For both, we used the binary C/C++ implementations. All runtime experiments were conducted on the same machine running Ubuntu 20.04.3 LTS with a total of 52 CPUs, 756 GB of memory and 4 NVIDIA GeForce RTX 3090 GPUs, each with 24 GB of memory. For our experiments, we restricted the number of CPUs to 1 and 8 cores and a single GPU using dedicated Docker containers. We took the mean of the runtime over three runs for each experiment.

**Fig. 2.**
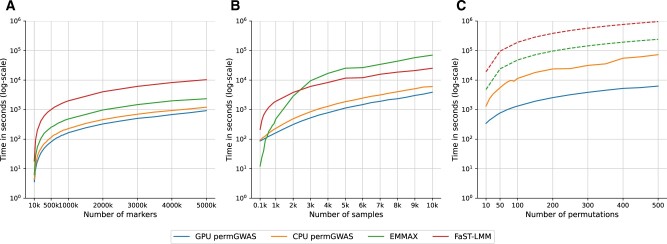
Runtime comparison of permGWAS versus EMMAX and FaST-LMM. Note that all axes are log-scaled. (**A**) Computational time as function of number of SNPs with fixed number of 1000 samples. (**B**) Computational time as function of number of samples with 10^6^ markers each. (**C**) Computational time as function of number of permutations with 1000 samples and 10^6^ markers each. Dashed lines for EMMAX and FaST-LMM are estimated based on the computational time for 1000 samples and 10^6^ markers times the number of permutations

First, we compared the runtime on environments with a single CPU and GPU. For this purpose, we fixed the number of samples to 1000 and varied the markers between 10^4^ and $5\times 10^{6}$ to evaluate the effect of an increasing number of SNPs. As summarized in Figure 2A, all models show a linear dependency with respect to the number of SNPs. permGWAS (the GPU and CPU version) outperforms both, the binary implementation of EMMAX and FaST-LMM. Our dockerized Python implementation of permGWAS is almost one order of magnitude faster than the C/C++ implementation of FaST-LMM (for 1000 samples and $5\times 10^{6}$ markers 0.33 and 2.8 h, respectively). This can be mainly explained due to the batch-wise computation of several univariate statistical tests simultaneously.

Next, to estimate the effect of the number of samples on the runtime, we fixed the number of SNPs to 10^6^ and varied the number of samples between 100 and 10^4^. In Figure 2B, we can observe that EMMAX outperforms all other comparison partners for sample sizes smaller than 500. However, the runtime increases quickly for larger samples sizes. Again permGWAS outperforms both comparison partners by at least one order of magnitude. Here, the runtime of the GPU version of permGWAS for 10^4^ samples and 10^6^ markers was approximately 1.7 h, while for FaST-LMM and EMMAX the runtime was more than 7 and 19 h, respectively.

Finally, to compare the runtime of the permutation-based approach, we fixed the number of samples to 1000 and the number of SNPs to 10^6^ and conducted between 10 and 500 permutations with permGWAS using a single GPU architecture versus a single CPU. Since EMMAX and FaST-LMM only perform one univariate test at a time and are not designed for permutation-based tests, we took the runtime for 1000 samples and 10^6^ markers from the previous experiment and estimated the runtime for permutations by multiplying with the number of permutations. This is just an estimate of the minimal runtime, since no data pre-processing and post-processing steps are included (e.g. preparing permuted phenotypes, merging result files to estimate adjusted *P*-values/thresholds). The advantage of the GPU architecture becomes most obvious when using permutations, as illustrated in Figure 2C. The GPU version of permGWAS is at least an order of magnitude faster than the CPU version of permGWAS. More importantly, permGWAS (GPU) is more than one order of magnitude faster than EMMAX and more than two orders of magnitude faster than FaST-LMM. Even for 1000 samples, 10^6^ SNPs and 500 permutations permGWAS (GPU) takes less than 1.8 h. In contrast, EMMAX would require at least more than 2.7 days, while FaST-LMM might take more than 11 days for 500 permutations. Results for environments with 8 cores are summarized in Supplementary Figure S1 and show similar results.

In addition, we re-analyzed over 500 *A.thaliana* phenotypes with 100 permutations each on a single GPU (Nvidia RTX A5000 with 24 GB RAM) in less than 8 days. The respective runtimes are shown in Supplementary Figure S2. Notable, for phenotypes with a sample size above 800 individuals, the 24 GB RAM weren’t sufficient and the analysis has been performed on an HPC environment allowing for additional RAM.

In summary, permGWAS is more efficient than the commonly used state-of-the-art LMMs, such as EMMAX and FaST-LMM, due to its tensor-based and batch-wise reformulation. Especially when performing GWAS with more than a few hundred of samples and permutations, EMMAX and FaST-LMM take several days to weeks to compute the results, while our implementation only needs a few hours. Although the GPU implementation of permGWAS is faster than the corresponding CPU implementation, the CPU version still outperforms existing methods.

#### 3.1.2 FDR for skewed phenotypes

**Fig. 3.**
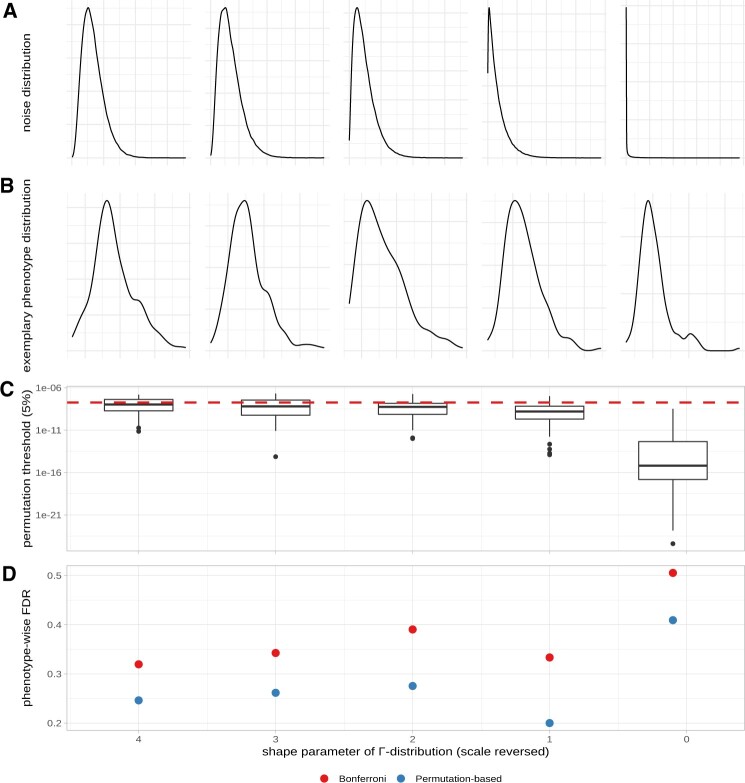
Simulated phenotypes with gamma-distributed noise. Shape parameters of the gamma distributions were set at 4, 3, 2, 1 and 0.1. (**A**) Shape of the gamma distribution. (**B**) Exemplary phenotypic value distribution for each shape parameter. (**C**) Permutation-based thresholds over 50 simulated phenotypes as box plots for each gamma shape parameter. Red dashed line illustrates the fixed Bonferroni significance threshold. (**D**) Phenotype-wise FDR for both the fixed Bonferroni significance threshold and the permutation-based significance threshold

Our simulations show that the phenotype-wise FDR increases, if the respective phenotypes become more skewed. Using a static Bonferroni threshold, the phenotype-wise FDR increases from 30% for slightly skewed phenotypes to 50% in the most extreme case (Fig. 3D). The latter means that in nearly all of the simulated phenotypes, not only true, but also false associations have been found (Supplementary Table S2).

Noticeably, the permutation-based threshold becomes more and more stringent, if the phenotypic distribution becomes more skewed (Fig. 3C), thereby controlling the phenotype-based FDR more reliable (Fig. 3D and Supplementary Table S2). Skewed phenotypic distributions will violate model assumptions, and associations with low *P*-values can arise randomly. This will get controlled by permutations that can account for model violations, as underlying assumptions are also violated in a model without genetic signal. Hence, permutations can control for false associations that arise through non-normal phenotypic distributions.

On the other hand, for normally distributed phenotypes, the permutation-based threshold is less stringent compared to the Bonferroni threshold (Supplementary Fig. S3C) and increases the power to recover true associations (Supplementary Fig. S3D). However, also slightly more false positives are detected. To summarize, simulations suggest that a permutation-based threshold is more flexible compared to a static Bonferroni threshold and will provide a higher power to detect true associations for normally distributed phenotypes, as well as control FDR for skewed phenotypes.

### 3.2 Permutation-based GWAS in *Arabidopsis thaliana*

**Fig. 4.**
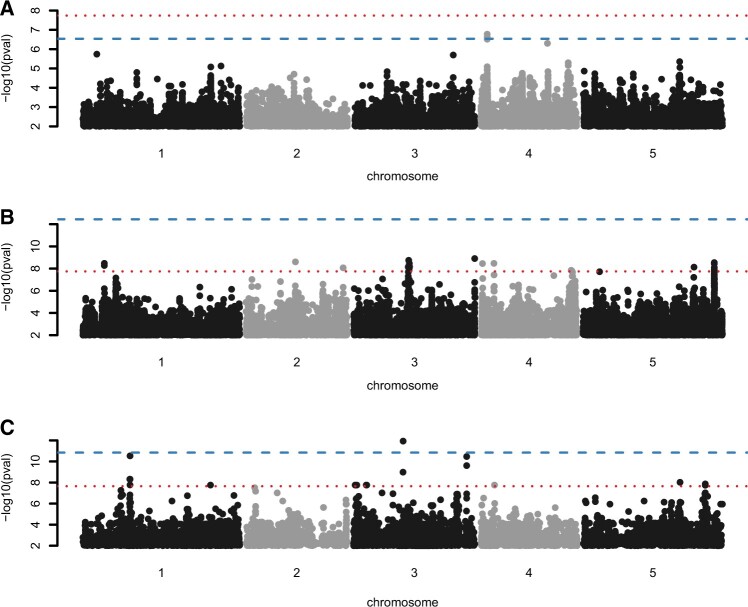
GWAS of three different *A.thaliana* phenotypes. Manhattan plots display the associations of all markers for the three phenotypes (**A**) #744 (https://arapheno.1001genomes.org/phenotype/744/), which is nearly normal distributed, (**B**) #118 (https://arapheno.1001genomes.org/phenotype/118/) and (**C**) #325 (https://arapheno.1001genomes.org/phenotype/325/). Where the two latter phenotypes are non-normally distributed. The Bonferroni threshold is denoted by a red horizontal dashed line and the respective permutation-based threshold by a horizontal blue line

After we highlighted the advantages of a permutation-based threshold with simulated data, we re-analyzed 516 real phenotypes that we have downloaded from the phenotypic data repository AraPheno (Seren *et al.*, 2016; Togninalli *et al.*, 2020). As expected for real data, many of these are non-normally distributed. Using the Shapiro–Wilk test on the phenotypic data, only 90 phenotypes had a *P*-value > 0.05, indicating a normal distribution (Supplementary File S1). As expected by our simulations, we observed a correlation between the phenotypic distribution and the calculated permutation-based threshold (Supplementary Fig. S4). All but two phenotypes that are normally distributed (Shapiro–Wilk test > 0.05) show a less stringent permutation-based threshold compared to the Bonferroni threshold (Supplementary Fig. S4 inset). In summary, for the 516 analyzed phenotypes, the permutation-based thresholds are 293 times more stringent and 223 less stringent compared to the Bonferroni threshold. Although, we do not know the ground truth of true and false associations for this data, permutation-based thresholds markedly reduce the overall number of associations, especially for skewed phenotypes. Comparing the 100 most skewed phenotypes (*P*-value from the Shapiro–Wilk test $<10^{-19}$), nearly all (96) show a significant association using the Bonferroni threshold, while only six of the most normal distributed phenotypes (*P*-value from the Shapiro–Wilk test > 0.02) have a significant association. Using the permutation-based threshold, these numbers change to 53 and 15, respectively (summary results of all analyzes can be found in Supplementary File S1). *A priori*, there is no reason why skewed phenotypes should more often show true associations. Therefore, the number of reported associations with the permutation-based threshold seems more realistic. In general, we can observe different scenarios: (1) for some cases, a less stringent permutation-based threshold will identify a significant signal that would not have been significant using the Bonferroni threshold (Fig. 4A). This scenario is true for 22 different phenotypes, especially if their phenotypic distribution is normal (Supplementary Fig. S5A); (2) We observed 123 cases, where the Bonferroni threshold would indicate significant associations, but the permutation-based threshold would rather assume that these are false positives (Fig. 4B) and (3) for another 111 cases, even after using a permutation-based threshold, skewed phenotypes show still significant associations (Fig. 4C). Most phenotypes that belong to scenario (2) or (3) are non-normally distributed (Supplementary Fig. S5B and C).

Although follow-up experiments would be needed to confirm that associations deemed positive in the scenarios (1) and (3) are true positives, anecdotally many of this candidates seem plausible.

### 3.3 Number of permutations and minor allele frequencies

In the previous paragraph, we emphasized the benefits of using a permutation-based threshold instead of a Bonferroni threshold. We performed 100 permutations for each phenotype, but more permutations could give a more accurate estimate of the threshold. To investigate the effect of the number of permutations on the permutation-based threshold, we performed additional permutations for two different *A.thaliana* phenotypes. One phenotype is nearly normally distributed, while the other is markedly skewed (Supplementary Fig. S6A and C). We performed 100, 200, 300, 400, 500 and 1000 permutations. Here the used 5% threshold is nearly identical and stable across the different number of permutations performed for both phenotypes (Supplementary Fig. S6B and D). Thus, our empirical results suggest that 100 permutations will give rise to a reliable estimate of the threshold and enable a fast analysis of many phenotypes and or huge data.

Next, we analyzed if minor allele frequency has an effect on false positives and the respective calculated permutation-based thresholds. It has been suggested that rare variants can easily associate with phenotypic extremes and thus that false positive associations of rare alleles are more prone in non-normally distributed phenotypes (Peloso *et al.*, 2016). If this is true, a permutation-based threshold should be able to account for excessive false associations of rare alleles. Using permGWAS with increasing minor allele filters and thereby excluding rare alleles from the analysis, the Bonferroni threshold is just reflecting the lower amount of markers tested, while the permutation-based threshold has a non-linear dependency. For normally distributed phenotypes, the change in the permutation-based threshold is similar to Bonferroni (Supplementary Fig. S7A), while for a skewed phenotype a clear effect of excluding rare alleles is observed. As an example, in the analysis of phenotype #372 (DOI: 10.21958/phenotype:372) from *A.thaliana*, the calculated permutation-based threshold increases from $10^{-16}$ if all markers are analyzed to $10^{-10}$ if only alleles with a minor allele count of at least 10 are considered (Supplementary Fig. S7B). For skewed phenotypes, the permutation-based threshold is clearly dependent on the allele frequency. permGWAS can compute and provide a distinct threshold for different allele frequencies that is—unlike Bonferroni—dependent on the phenotpic distribution and not the amount of markers tested.

## 4 Conclusions

We introduced permGWAS, an efficient LMM for GWAS with population structure correction and permutation-based significance thresholds that can reliably control false positives for phenotypes with skewed distributions. Our method uses a 4D tensor reformulation of a LMM using a permutation strategy proposed by Westfall–Young (Westfall and Young, 1993) to compute univariate association tests batch-wise, on both modern multi-core and GPU environments. We compared permGWAS in terms of runtime with EMMAX (Kang *et al.*, 2010) and FaST-LMM (Lippert *et al.*, 2011), two state-of-the-art LMMs. We could show that permGWAS outperformed both models in terms of computational and statistical performance. Especially, permGWAS is highly efficient in a permutation-based setting, due to the 4D tensor reformulation and the available GPU support [2 h for permGWAS (GPU) versus several days for EMMAX and FaST-LMM for 1000 samples, 10^6^ markers and 500 permutations]. These reformulations enable performing permutation-based thresholds in practice.

We demonstrated through simulations and the re-analyses of publicly available data from the model plant species *A.thaliana* that the use of a permutation-based threshold has many advantages compared to the classically used Bonferroni threshold. Bonferroni correction is thought as a very conservative way to control false positives in GWAS, and indeed for normal distributed phenotypes, we could show that the permutation-based threshold is less stringent and can identify more true positive associations. Further, for non-normally distributed phenotypes, as often observed in biological data, the permutation-based threshold is quite often even more stringent. Here, our data suggest that we could reliably control false positives under those scenarios. A sensible next step would be to investigate how to extend permGWAS to Generalized LMMs, e.g. to properly handle the analysis of binary traits. To summarize, we highlight that the use of permutation-based thresholds should be considered the default choice in any GWAS and with permGWAS we provide the tool to enable this.

## Funding

This paper was published as part of a special issue financially supported by ECCB2022. The work was supported in parts by funds of the Federal Ministry of Education and Research (BMBF), Germany [01—S21038B, D.G.G.].

*Conflict of Interest*: none declared.

## Supplementary Material

btac455_Supplementary_Data

## References

[btac455-B1] Arouisse B. et al (2020) Imputation of 3 million SNPs in the Arabidopsis regional mapping population. Plant J., 102, 872–882. 31856318 10.1111/tpj.14659 PMC7318218

[btac455-B2] Atwell S. et al (2010) Genome-wide association study of 107 phenotypes in *Arabidopsis thaliana* inbred lines. Nature, 465, 627–631. 20336072 10.1038/nature08800 PMC3023908

[btac455-B3] Bonferroni C. (1936) Teoria statistica delle classi e calcolo delle probabilita. Pubblicazioni del R Istituto Superiore di Scienze Economiche e Commericiali di Firenze, 8, 3–62.

[btac455-B4] Che R. et al (2014) An adaptive permutation approach for genome-wide association study: evaluation and recommendations for use. BioData Min., 7, 9. 24976866 10.1186/1756-0381-7-9 PMC4070098

[btac455-B5] Freudenthal J.A. et al (2019) GWAS-flow: a GPU accelerated framework for efficient permutation based genome-wide association studies. *BioRxiv*, 783100.

[btac455-B6] Grimm D.G. et al (2017) easyGWAS: a cloud-based platform for comparing the results of genome-wide association studies. Plant Cell, 29, 5–19. 27986896 10.1105/tpc.16.00551 PMC5304348

[btac455-B7] Gumpinger A.C. et al; International Headache Genetics Consortium. (2021) Network-guided search for genetic heterogeneity between gene pairs. Bioinformatics, 37, 57–65. 32573681 10.1093/bioinformatics/btaa581 PMC8034561

[btac455-B8] Harris C.R. et al (2020) Array programming with NumPy. Nature, 585, 357–362. 32939066 10.1038/s41586-020-2649-2 PMC7759461

[btac455-B9] Hayes B.J. et al (2009) Increased accuracy of artificial selection by using the realized relationship matrix. Genet. Res. (Camb.), 91, 47–60. 19220931 10.1017/S0016672308009981

[btac455-B10] Kang H.M. et al (2008) Efficient control of population structure in model organism association mapping. Genetics, 178, 1709–1723. 18385116 10.1534/genetics.107.080101 PMC2278096

[btac455-B11] Kang H.M. et al (2010) Variance component model to account for sample structure in genome-wide association studies. Nat. Genet., 42, 348–354. 20208533 10.1038/ng.548 PMC3092069

[btac455-B12] Korte A. et al (2012) A mixed-model approach for genome-wide association studies of correlated traits in structured populations. Nat. Genet., 44, 1066–1071. 22902788 10.1038/ng.2376 PMC3432668

[btac455-B13] Lippert C. et al (2011) Fast linear mixed models for genome-wide association studies. Nat. Methods, 8, 833–835. 21892150 10.1038/nmeth.1681

[btac455-B14] Lippert C. et al (2014) LIMIX: genetic analysis of multiple traits. *BioRxiv.*

[btac455-B15] Llinares-López F. et al (2015) Genome-wide detection of intervals of genetic heterogeneity associated with complex traits. Bioinformatics, 31, i240–i249. 26072488 10.1093/bioinformatics/btv263 PMC4559912

[btac455-B16] Loh P.-R. et al (2015) Efficient Bayesian mixed-model analysis increases association power in large cohorts. Nat. Genet., 47, 284–290. 25642633 10.1038/ng.3190 PMC4342297

[btac455-B17] McKinney W. et al (2011) pandas: a foundational Python library for data analysis and statistics. Python High Performance Sci. Comput., 14, 1–9.

[btac455-B18] Paszke A. et al (2019) PyTorch: an imperative style, high-performance deep learning library. Adv. Neural Inf. Process. Syst., 32.

[btac455-B19] Peloso G.M. et al (2016) Phenotypic extremes in rare variant study designs. Eur. J. Hum. Genet., 24, 924–930. 26350511 10.1038/ejhg.2015.197 PMC4867440

[btac455-B20] Purcell S. et al (2007) PLINK: a tool set for whole-genome association and population-based linkage analyses. Am. J. Hum. Genet., 81, 559–575. 17701901 10.1086/519795 PMC1950838

[btac455-B21] Seren Ü. et al (2016). AraPheno: a public database for *Arabidopsis thaliana* phenotypes. Nucleic Acids Res., 45, D1054–D1059. 27924043 10.1093/nar/gkw986 PMC5210660

[btac455-B22] Shen X. , Rönnegård L. (2013) Issues with data transformation in genome-wide association studies for phenotypic variability. F1000Res, 2, 200. [pmid]. 24555098 10.12688/f1000research.2-200.v1 PMC3869493

[btac455-B23] Storey J.D. , Tibshirani R. (2003) Statistical significance for genomewide studies. Proc. Natl. Acad. Sci. USA, 100, 9440–9445. 12883005 10.1073/pnas.1530509100 PMC170937

[btac455-B24] Sun X. et al (2013) What is the significance of difference in phenotypic variability across SNP genotypes? Am. J. Hum. Genet., 93, 390–397. 23910463[pmid]. 23910463 10.1016/j.ajhg.2013.06.017 PMC3738833

[btac455-B25] Swiel Y. et al (2022) FPGA acceleration of GWAS permutation testing. *bioRxiv.* 10.1093/bioadv/vbaf145 PMC12237511 40630501

[btac455-B26] The 1001 Genomes Consortium. (2016) 1,135 genomes reveal the global pattern of polymorphism in *Arabidopsis thaliana*. Cell, 166, 481–491. 27293186 10.1016/j.cell.2016.05.063 PMC4949382

[btac455-B27] Todesco M. et al (2020) Massive haplotypes underlie ecotypic differentiation in sunflowers. Nature, 584, 602–607. 32641831 10.1038/s41586-020-2467-6

[btac455-B28] Togninalli M. et al (2020) AraPheno and the AraGWAS Catalog 2020: a major database update including RNA-seq and knockout mutation data for *Arabidopsis thaliana*. Nucleic Acids Res., 48, D1063–D1068. 31642487 10.1093/nar/gkz925 PMC7145550

[btac455-B29] Virtanen P. et al; SciPy 1.0 Contributors. (2020) Scipy 1.0: fundamental algorithms for scientific computing in python. Nat. Methods, 17, 261–272. 32015543 10.1038/s41592-019-0686-2 PMC7056644

[btac455-B30] Westfall P.H. , Young S.S. (1993) Resampling-Based Multiple Testing: Examples and Methods for p-Value Adjustment. Vol. 279. John Wiley & Sons, New York.

