# Correction to: Efficient permutation-based genome-wide association studies for normal and skewed phenotypic distributions

**DOI:** 10.1093/bioinformatics/btac690

**Published:** 2022-10-22

This is a correction to: Maura John, Markus J. Ankenbrand, Carolin Artmann, Jan A. Freudenthal, Arthur Korte, and Dominik G. Grimm, Efficient permutation-based genome-wide association studies for normal and skewed phenotypic distributions, *Bioinformatics*, Volume 38, Issue Supplement_2, September 2022, Pages ii5–ii12, https://doi.org/10.1093/bioinformatics/btac455

In the originally published version of this manuscript, the incorrect Supplementary Data was erroneously included. This error has been corrected online.

## Supplementary Material

btac690_Supplementary_Data

