# Supplementary material for: Efficient permutation-based genome-wide association studies for normal and skewed phenotypic distributions
Source: Bioinformatics. 2022 Sep 18;38(Suppl 2):ii5–ii12. doi: 10.1093/bioinformatics/btac455 (PMC9486594; doi:10.1093/bioinformatics/btac455)
Supplement: btac455_Supplementary_Data [file btac455_supplementary_data.pdf]

SUPPLEMENT TO "BAYESIAN MODELING OF SPATIAL MOLECULAR PROFILING DATA VIA GAUSSIAN PROCESS"

By Qiwei Li[†,*], Minzhe Zhang[†], Yang Xie, and Guanghua Xiao[*]

1. MCMC Algorithm. The model parameter space consists of $(\boldsymbol{H}, \boldsymbol{\phi}, \boldsymbol{\Lambda}, \boldsymbol{\gamma}, \boldsymbol{l})$, where $\boldsymbol{H} = \{\eta_{ij}, i = 1, \ldots, n, j = 1, \ldots, p\}$ is the extra zero (i.e. false or structural zero) indicator matrix, $\boldsymbol{\phi} = \{\phi_j, j = 1, \ldots, p\}$ is the collection of dispersion parameters for all genes, $\boldsymbol{\Lambda} = \{\lambda_{ij}, i = 1, \ldots, n, j = 1, \ldots, p\}$ is the collection of normalized expression levels, $\boldsymbol{\gamma} = \{\gamma_j, j = 1, \ldots, p\}$ is the SV gene indicator vector, and $\boldsymbol{l}$ is the kernel parameters. We start by writing the full posterior,

$$p(\boldsymbol{H}, \boldsymbol{\phi}, \boldsymbol{\Lambda}, \boldsymbol{\gamma}, \boldsymbol{l}|\boldsymbol{Y}) \quad \propto \quad f(\boldsymbol{Y}|\boldsymbol{H}, \boldsymbol{\phi}, \boldsymbol{\Lambda})p(\boldsymbol{H})p(\boldsymbol{\phi})p(\boldsymbol{\Lambda}|\boldsymbol{H}, \boldsymbol{\gamma}, \boldsymbol{l})p(\boldsymbol{l}|\boldsymbol{\gamma})p(\boldsymbol{\gamma}).$$

According to Section 2.1 in the main text, we can compute the likelihood and priors as

$$\begin{aligned} f(\boldsymbol{Y}|\boldsymbol{H}, \boldsymbol{\phi}, \boldsymbol{\Lambda}) &= \prod_{i=1}^{n} \prod_{\{j:\eta_{ij}=0\}} \mathrm{NB}(y_{ij}; s_i\lambda_{ij}, \phi_j), \\ p(\boldsymbol{H}) &= \prod_{i=1}^{n} \prod_{j=1}^{p} \text{Be-Bern}(\eta_{ij}; a_\pi, b_\pi), \\ p(\boldsymbol{\phi}) &= \prod_{j=1}^{p} \mathrm{Ga}(\phi_j; a_\phi, b_\phi), \end{aligned}$$

and according to Section 2.2, we can calculate the rest of priors as

$$\begin{aligned} p(\boldsymbol{\Lambda}|\boldsymbol{H}, \boldsymbol{\gamma}, \boldsymbol{l}) &= \prod_{j=1}^{p} p(\log \boldsymbol{\lambda}_j|\gamma_j, l_j), \\ p(\boldsymbol{\gamma}) &= \prod_{j=1}^{p} \text{Be-Bern}(\gamma_j; a_\omega, b_\omega), \\ p(\boldsymbol{l}|\boldsymbol{\gamma}) &= \prod_{\{j:\gamma_j=1\}} \mathrm{Ga}(l_j; a_l, b_l), \end{aligned}$$

where the explicit formula of $p(\log \boldsymbol{\lambda}_j|\gamma_j, l_j)$ is given in Equation (5) in the main text. The p.m.f.'s or p.d.f.'s of the involved common distributions are given below:

$$\begin{aligned} \text{If} \quad x \sim \mathrm{NB}(\mu, \phi), \quad \text{then} \quad p(x) &= \frac{\Gamma(x+\phi)}{x!\Gamma(\phi)} \left(\frac{\phi}{\mu+\phi}\right)^{\phi} \left(\frac{\mu}{\mu+\phi}\right)^{x}, \\ \text{If} \quad x \sim \text{Be-Bern}(a, b), \quad \text{then} \quad p(x) &= \frac{1}{a+b} \frac{\Gamma(a+x)\Gamma(b+1-x)}{\Gamma(a)\Gamma(b)}, \\ \text{If} \quad x \sim \mathrm{Ga}(a, b), \quad \text{then} \quad p(x) &= \frac{b^a}{\Gamma(a)} x^{a-1} \exp(-bx). \end{aligned}$$

---

[*]To whom correspondence should be addressed
[†]These authors contributed equally to this work

Identifying SV genes through the selection vectors $\boldsymbol{\gamma}$ is our main interest. To serve this purpose, a MCMC algorithm is designed based on Metropolis search variable selection algorithms (George and McCulloch 1997, Brown *et al.* 1998). As discussed in Section 2.2 in the main text, we have integrated out the mean and covariance scaling factor. This step helps us speed up the MCMC convergence and improve the estimation of $\boldsymbol{\gamma}$. At each MCMC iteration, we perform the following steps:

Update of zero-inflation indicator $\boldsymbol{H}$: We update each $\boldsymbol{\eta}_j, j = 1, \ldots, p$ separately. For gene j, we update each false zero indicator $\eta_{ij}, i = 1, \ldots, n$ that corresponds to $y_{ij} = 0$ using the Gibbs sampler,

$$\begin{aligned} p(\eta_{ij}|\cdot) &\propto \text{NB}(y_{ij}; s_i\lambda_{ij}, \phi_j) \times p(\log \boldsymbol{\lambda}_j|\gamma_j, l_j) \times \text{Be-Bern}(\eta_{ij}; a_\pi, b_\pi) \\ \eta_{ij}|\cdot &\sim \text{Bern}\left(\frac{p(\eta_{ij} = 1|\cdot)}{p(\eta_{ij} = 0|\cdot) + p(\eta_{ij} = 1|\cdot)}\right) \end{aligned}$$

Note that $p(\log \boldsymbol{\lambda}_j|\gamma_j, l_j)$ might canceled out due to the limited contribution of a single location towards the calculation the entire normalized expression level across the space.

Update of dispersion parameter $\boldsymbol{\phi}$: We update each $\phi_j, j = 1, \ldots, p$ separately by using a random walk Metropolis-Hastings (RWMH) algorithm. We first propose a new ${\phi_j}^*$, of which logarithmic value is generated from $\text{N}\left(\log \phi_j, \tau_\phi^2\right)$ and then accept the proposed value ${\phi_j}^*$ with probability $\min(1, m_{\text{MH}})$, where the Hastings ratio is

$$m_{\text{MH}} = \prod_{\{i:\eta_{ij}=0\}} \frac{\text{NB}(y_{ij}; s_i\lambda_{ij}, \phi_j^*)}{\text{NB}(y_{ij}; s_i\lambda_{ij}, \phi_j)} \frac{\text{Ga}(\phi_j^*; a_\phi, b_\phi)}{\text{Ga}(\phi_j; a_\phi, b_\phi)} \frac{J\left(\phi_j \leftarrow \phi_j^*\right)}{J\left(\phi_j^* \leftarrow \phi_j\right)}.$$

We use $J(\cdot \leftarrow \cdot)$ to denote the proposal density. Note that the proposal density ratio cancels out for this RWMH update.

Update of normalized gene expression levels $\boldsymbol{\Lambda}$: We update each $\boldsymbol{\lambda}_j, j = 1, \ldots, p$ separately. For gene j, we update its normalized gene expression at location $\boldsymbol{t}_{i,\cdot}, i = 1, \ldots, n$ sequentially by using the RWMH algorithm. We first propose a new λ_{ij}^* from $\text{N}\left(\lambda_{ij}, \tau_\lambda^2\right)$, and then accept the proposed value with probability $\min(1, \text{m}_{\text{MH}})$, where the Hastings ratio is

$$\text{m}_{\text{MH}} = \frac{\text{NB}(y_{ij}; s_i\lambda_{ij}^*, \phi_j)}{\text{NB}(y_{ij}; s_i\lambda_{ij}, \phi_j)} \frac{p(\log \boldsymbol{\lambda}_j^*|\gamma_j, l_j)}{p(\log \boldsymbol{\lambda}_j|\gamma_j, l_j)} \frac{J\left(\lambda_{ij} \leftarrow \lambda_{ij}^*\right)}{J\left(\lambda_{ij}^* \leftarrow \lambda_{ij}\right)}.$$

Note that the proposal density ratio cancels out for this RWMH update. If $\eta_{ij} = 1$ (corresponding to a false zero count), then the first term also cancels out.

Joint update of SV gene indicator $\boldsymbol{\gamma}$ and kernel parameter $\boldsymbol{l}$: We perform a between-model step to update these two groups of parameters jointly since $\boldsymbol{l}$ depends on $\boldsymbol{\gamma}$. This is done via an *add-delete* algorithm. In this approach, a new candidate vector, say $\boldsymbol{\gamma}^*$, is generated by randomly choosing an entry of $\boldsymbol{\gamma}$, say j, and changing its value to $\gamma_j^* = 1 - \gamma_j$. Then, this proposed move is accepted with probability $\min(1, \text{m}_{\text{MH}})$, where the Hastings ratio is

$$\text{m}_{\text{MH}} = \frac{p(\log \boldsymbol{\lambda}_j|\gamma_j^*, l_j^*)}{p(\log \boldsymbol{\lambda}_j|\gamma_j, l_j)} \frac{\left(\text{Ga}(l_j^*; a_l, b_l)\right)^{\gamma_j^*}}{\left(\text{Ga}(l_j; a_l, b_l)\right)^{\gamma_j}} \frac{p(\boldsymbol{\gamma}^*)}{p(\boldsymbol{\gamma})} \frac{J\left(l_j \leftarrow l_j^*|\gamma_j \leftarrow \gamma_j^*\right)}{J\left(l_j^* \leftarrow l_j|\gamma_j^* \leftarrow \gamma_j\right)} \frac{J\left(\gamma_j \leftarrow \gamma_j^*\right)}{J\left(\gamma_j^* \leftarrow \gamma_j\right)},$$

where we use $J(\cdot \leftarrow \cdot)$ to denote the proposal density for the selected move. For the *add* case, we propose a new l_j^*, of which logarithmic value is drawn from a truncated normal distribution $\mathrm{N}_{[a_l,b_l]}\left(\log(t^{\min}/2),(10\tau_l)^2\right)$. The third ratio equals to a_ω/b_ω for the *add* step and the reciprocal for the *delete* step. The second to the last proposal density ratio equals to

$$\begin{cases} 1/\mathrm{N}(\log l_j^*;\log(t^{\min}/2),(10\tau_l)^2) & \text{for } \textit{add} \\ \mathrm{N}(\log l_j;\log(t^{\min}/2),(10\tau_l)^2) & \text{for } \textit{delete} \end{cases},$$

while the last proposal density ratio equals to one.

Update of kernel parameter $\boldsymbol{l}$: We perform a within-model step to update each l_j of which $\gamma_j = 1$ separately by using the RWMH algorithm. Specifically, we propose a new $l_j{}^*$, of which logarithmic value is sampled from a truncated normal distribution $\mathrm{N}_{[a_l,b_l]}\left(\log l_j, \tau_l^2\right)$, and then accept the proposed value $l_j{}^*$ with probability $\min(1, m_{\mathrm{MH}})$, where the Hastings ratio is

$$m_{\mathrm{MH}} \quad = \quad \frac{p(\log \boldsymbol{\lambda}_j|\gamma_j, l_j^*)}{p(\log \boldsymbol{\lambda}_j|\gamma_j, l_j)} \frac{\mathrm{Ga}(l_j^*; a_l, b_l)}{\mathrm{Ga}(l_j; a_l, b_l)} \frac{J\left(l_j \leftarrow l_j^*\right)}{J\left(l_j^* \leftarrow l_j\right)}.$$

Note that the proposal density ratio cancels out.

2. Sensitivity Analysis. We examined the model sensitivity with respect to the choice of hyperparameters b_σ and h. The numerical summary shown in Table S3 demonstrates that our approach was considerably insensitive to the hyperparameter settings.

The hyperparameters b_σ and h are related to the covariance term in the multivariate student's t mixture model shown in Equation (5) in the main text. We considered a range of (b_σ, h) settings as $b_\sigma \in \{1/4, 1, 4, 16\}$ and $h \in \{1, 10, 100\}$. Then we applied BOOST-GP with different combinations of (b_σ, h) to those datasets simulated from the MOB pattern and zero-inflation setting (i.e. 30% false zeroes). We reported the averaged AUCs over ten replicated in Table S3, based on two posterior summarizations: Bayes factors and marginal posterior probabilities of inclusion. The AUCs remained stable under different choices of (b_σ, h), while increasing b_σ resulted in a decreased ability to identify the SV genes. We suggest to set $b_\sigma = 1$ and $h = 10$ as BOOST-GP default values as this combination achieved the best result.

3. Moran's $\boldsymbol{I}$ Spatial Autocorrelation Test. The original Moran's I (Moran 1950) is defined as

$$\text{Moran's } I = \frac{n}{\sum_{i=1}^n \sum_{i'=1}^n w_{ii'}} \frac{\sum_{i=1}^n \sum_{i'=1}^n w_{ii'}(z_i - \bar{z})(z_{i'} - \bar{z})}{\sum_{i=1}^n (z_i - \bar{z})^2},$$

where n is the number of locations (e.g. cells or spots) indexed by i or i', z_i is the variable of interest measured on location i, $\bar{z} = \sum_{i=1}^n z_i/n$ is the mean, and $\boldsymbol{W} = [w_{ii'}]_{n\times n}$ is a matrix of spatial weight with zeros on the diagonal (i.e. $w_{ii'} = 0$). In our real data analysis, we assumed $w_{ii'}$ was binary with $w_{ii'} = 1$ indicating locations i and i' are neighbors and $w_{ii'} = 0$ otherwise. We evaluated two scenarios where neighbors of a location were defined as its nearby points in the surrounding 3×3 (eight-connected) or 5×5 grid (24-connected).

For each identified SV gene, we conducted the Moran's I spatial autocorrelation test (Li *et al.* 2007) as follows. We first calculated Moran's I_{obs} based on the observed data for each individual gene j. In particular, we let $\boldsymbol{z} = (\log \lambda_{1j}, \ldots, \log \lambda_{nj})$ be the log normalized expression levels of all spots, where each $\log \lambda_{ij}$ was the posterior mean estimated from the MCMC samples. Then, we

performed 50 random permutations on $\boldsymbol{z}$ and calculated their Moran's I. We denoted their average value as Moran's I_{null}. Finally, we reported the difference between Moran's I_{obs} and Moran's I_{null},

$$\text{Moran's } I = \text{Moran's } I_{\text{obs}} - \text{Moran's } I_{\text{null}}.$$

4. Supplementary Figures and Tables. We provide the supplementary figures and tables here.

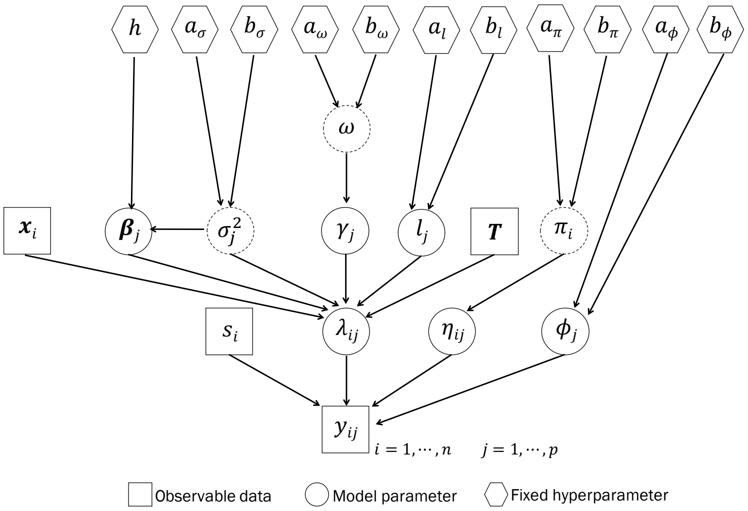

FIG S1. *A graphical representation of BOOST-GP for identifying SV genes. Nodes in square, circle, and hexagon refer to observable data, model parameter, and fixed hyperparameter, respectively. Circles with dashed outline indicate nuisance parameters that are integrated out in BOOST-GP. The link between two nodes represents a direct probabilistic dependence.*

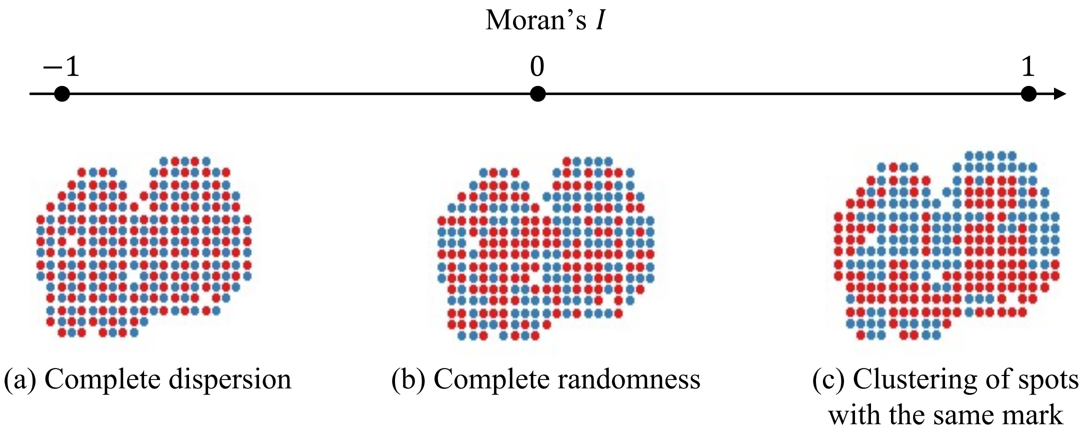

FIG S2. *An illustration of the spatial autocorrelation measurement Moran's I. (a) The blue and red spots are perfectly dispersed, corresponding to a Moran's* $I = -1$. *(b) A random arrangement of spot colors would give a Moran's* $I = 0$. *(c) The blue and red spots tend to stacked, resulting in a Moran's* $I \to 1$

TABLE S1

A hierarchical formulation of BOOST-GP for identifying SV genes.

Hierarchical model:

$$y_{ij}|s_i,\eta_{ij},\lambda_{ij} \overset{ind}{\sim} \eta_{ij}\,\mathrm{I}(y_{ij}=0)+(1-\eta_{ij})\,\mathrm{NB}(s_i\lambda_{ij},\phi_j), \quad i=1,\ldots,n$$

$$\log\boldsymbol{\lambda}_j|\gamma_j,l_j,\boldsymbol{\beta}_j,\sigma_j^2 \overset{ind}{\sim} \begin{cases}\mathrm{MN}(\boldsymbol{x}_i^\top\boldsymbol{\beta}_j,\sigma_j^2\boldsymbol{I}) & \text{if } \gamma_j=0\\ \mathrm{MN}(\boldsymbol{x}_i^\top\boldsymbol{\beta}_j,\sigma_j^2\boldsymbol{K}(\boldsymbol{T})) & \text{if } \gamma_j=1\end{cases}$$

False zero indicator prior:

$$\eta_{ij}|\pi_i \sim \mathrm{Bern}(\pi_i)$$
$$\pi_i \sim \mathrm{Be}(a_\pi,b_\pi)$$

Negative binomial dispersion prior:

$$\phi_j \sim \mathrm{Ga}(a_\phi,b_\phi)$$

Spatially variable (SV) gene indicator prior:

$$\gamma_j|\omega \sim \mathrm{Bern}\,(\omega)$$
$$\omega \sim \mathrm{Be}(a_\omega,b_\omega)$$

Characteristic length-scale prior:

$$l_j \sim \mathrm{Ga}(a_l,b_l)$$

Covariate coefficient prior:

$$\boldsymbol{\beta}_j|\sigma_j^2 \sim \mathrm{MN}(\mathbf{0},h\sigma_j^2\boldsymbol{I})$$

Scaling factor prior:

$$\sigma_j^2 \sim \mathrm{IG}(a_\sigma,b_\sigma)$$

Fixed hyperparameters:

$$a_\pi,b_\pi,a_\phi,b_\phi,a_\omega,b_\omega,a_l,b_l,h,a_\sigma,b_\sigma$$

Table S2

Simulation study: The averaged MCCs, with standard deviations in parentheses, achieved by BOOST-GP, SPARK, and SpatialDE under different scenarios in terms of spatial pattern and count generating process.

	Non-zero-inflation setting (i.e. 0% false zeros)			
	Spot pattern	Linear pattern	MOB pattern	BC pattern
SpatialDE	0.643 (0.104)	**0.810** (0.123)	0.213 (0.163)	0.416 (0.219)
SPARK	**0.770** (0.121)	0.425 (0.070)	**0.796** (0.061)	0.595 (0.054)
BOOST-GP	0.686 (0.111)	0.801 (0.121)	0.698 (0.090)	**0.723** (0.073)

	Zero-inflation setting (i.e. 30% false zeros)			
	Spot pattern	Linear pattern	MOB pattern	BC pattern
SpatialDE	0.000 (0.000)	0.000 (0.000)	0.000 (0.000)	0.000 (0.000)
SPARK	0.062 (0.103)	0.347 (0.155)	0.062 (0.103)	0.044 (0.114)
BOOST-GP	**0.237** (0.145)	**0.543** (0.073)	**0.208** (0.150)	**0.206** (0.192)

Table S3

Sensitivity analysis: The averaged AUCs, with standard deviations in parentheses, achieved by BOOST-GP with different hyperparameter choices under the scenario with the MOB pattern and zero-inflation setting (i.e. 30% *false zeros).*

	Based on the Bayes factors			
	$b_\sigma = 1/4$	$b_\sigma = 1$	$b_\sigma = 4$	$b_\sigma = 16$
$h = 1$	0.881 (0.036)	0.875 (0.041)	0.857 (0.041)	0.799 (0.052)
$h = 10$	0.865 (0.041)	**0.897** (0.045)	0.854 (0.034)	0.815 (0.068)
$h = 100$	0.871 (0.049)	0.896 (0.058)	0.865 (0.045)	0.814 (0.051)

	Based on the marginal posterior probabilities of inclusion			
	$b_\sigma = 1/4$	$b_\sigma = 1$	$b_\sigma = 4$	$b_\sigma = 16$
$h = 1$	0.856 (0.042)	0.820 (0.036)	0.793 (0.055)	0.684 (0.060)
$h = 10$	0.855 (0.048)	**0.893** (0.057)	0.791 (0.058)	0.691 (0.075)
$h = 100$	0.843 (0.049)	0.891 (0.058)	0.815 (0.061)	0.731 (0.059)

References.

Brown, P. J., Vannucci, M., and Fearn, T. (1998). Multivariate Bayesian variable selection and prediction. *Journal of the Royal Statistical Society: Series B (Statistical Methodology)* **60**, 3, 627–641.

George, E. I. and McCulloch, R. E. (1997). Approaches for Bayesian variable selection. *Statistica Sinica* 339–373.

Li, H., Calder, C. A., and Cressie, N. (2007). Beyond Moran's I: Testing for spatial dependence based on the spatial autoregressive model. *Geographical Analysis* **39**, 4, 357–375.

Moran, P. A. (1950). Notes on continuous stochastic phenomena. *Biometrika* **37**, 1/2, 17–23.
