# Supplementary material for: Correction to: Efficient permutation-based genome-wide association studies for normal and skewed phenotypic distributions
Source: Bioinformatics. 2022 Oct 22;38(22):5149. doi: 10.1093/bioinformatics/btac690 (PMC9665862; doi:10.1093/bioinformatics/btac690)
Supplement: btac690_Supplementary_Data [file btac690_supplementary_data.pdf]

Supplementary Information

Efficient Permutation-based Genome-wide Association Studies for Normal and Skewed Phenotypic Distributions

Maura John[1,2,*], Markus J Ankenbrand[3], Carolin Artmann[3], Jan A Freudenthal[3], Arthur Korte[3,*,§] and Dominik G Grimm[1,2,4,§]

[1]Technical University of Munich, Campus Straubing for Biotechnology and Sustainability, Bioinformatics, 94315 Straubing, Germany
[2]Weihenstephan-Triesdorf University of Applied Sciences, Bioinformatics, 94315 Straubing, Germany
[3]Center for Computational and Theoretical Biology, University of Würzburg, 97078 Würzburg, Germany
[4]Technical University of Munich, Department of Informatics, 85748 Garching, Germany

[*] Corresponding authors: arthur.korte@uni-wuerzburg.de, maura.john@hswt.de
[§] These authors contributed equally.

Author Contributions: AK and DGG conceived the study and supervised the project. MJ developed the mathematical framework. MJ implemented the model with input from JAF. MJA and CA simulated the phenotypes. MJ and MJA conducted the experiments. MJ, MJA, AK and DGG analyzed the results. MJ, AK and DGG wrote the paper with the contribution of all authors.

Contents

1 Supplementary Tables 2

2 Supplementary Figures 4

3 Supplementary Files 10

1 Supplementary Tables

Supplementary Table 1: Mathematical Symbols and Notations

n	number of samples
m	number of markers
c	number of fixed effects
b	number of batches
q	number of permutations
σ_g^2	extend of the genetic variance
σ_e^2	extend of the residual variance
α	significance threshold
δ	corrected significance threshold for FWER
δ^*	optimal significance threshold for FWER
$\delta_b^* = \frac{\alpha}{m}$	Bonferroni adjusted significance threshold
T_j	random variable for observed test statistic of the jth marker
${}^{(k)}t_j$	permutation test statistic of jth marker and k^{th} permutation
p_j	permutation based p-value of the jth marker
$\mathbb{1}$	indicator function, taking the value 1 if the argument is true and 0 otherwise
${}^{(k)}t_{max} = \max\limits_{j \in \{1,\ldots,m\}} {}^{(k)}t_j$	maximal test statistic of kth permutation
$\widetilde{p}_j$	adjusted p-value for Westfall-Young permutation testing
${}^{(k)}p_{min}$	minimal p-value of kth permutation
RSS_0	residual sum of squares of the null model
$\boldsymbol{y} \in \mathbb{R}^n$	vector of observed phenotypic values
$\boldsymbol{u} \in \mathbb{R}^n$	vector of random effects
$\boldsymbol{x}_j \in \mathbb{R}^n$	vector containing the jth SNP
$\boldsymbol{\epsilon} \in \mathbb{R}^n$	vector of residual effects
$\boldsymbol{\beta} \in \mathbb{R}^c$	vector of fixed effects coefficients
$\boldsymbol{X} \in \mathbb{R}^{n\times c}$	matrix of fixed effects
$\boldsymbol{K} \in \mathbb{R}^{n\times n}$	genetic relationship matrix
$\boldsymbol{I} \in \mathbb{R}^{n\times n}$	identity matrix of dimension n
$\boldsymbol{V} \in \mathbb{R}^{n\times n}$	variance-covariance matrix $\sigma_g^2\boldsymbol{K} + \sigma_e^2\boldsymbol{I}$
$\boldsymbol{C} \in \mathbb{R}^{n\times n}$	lower triangle matrix with $\boldsymbol{C}\boldsymbol{C}^T = \boldsymbol{V}$
$\boldsymbol{X}_j \in \mathbb{R}^{n\times c}$	matrix of fixed effects, containing a column of ones, the covariates and the jth SNP
$\boldsymbol{X}_j^b \in \mathbb{R}^{b\times n\times c}$	3D tensor containing $\boldsymbol{X}_j, \ldots, \boldsymbol{X}_{j+b-1}$
$\boldsymbol{Y}^b \in \mathbb{R}^{b\times n\times 1}$	3D tensor containing b copies of $\boldsymbol{y}$
$\boldsymbol{C}^b \in \mathbb{R}^{b\times n\times n}$	3D tensor containing b copies of $\boldsymbol{C}$
$\widetilde{\boldsymbol{X}}_j^b \in \mathbb{R}^{b\times n\times c}$	3D tensor containing the transformed data $\left(\boldsymbol{C}^b\right)^{-1}\boldsymbol{X}_j^b$
$\widetilde{\boldsymbol{Y}}^b \in \mathbb{R}^{b\times n\times 1}$	3D tensor containing the transformed data $\left(\boldsymbol{C}^b\right)^{-1}\boldsymbol{Y}^b$
$\left(\widetilde{\boldsymbol{X}}_j^b\right)^T \in \mathbb{R}^{b\times c\times n}$	3D tensor containing the transposed matrices $\left(\boldsymbol{C}^{-1}\boldsymbol{X}_j\right)^T, \ldots, \left(\boldsymbol{C}^{-1}\boldsymbol{X}_{j+b-1}\right)^T$
$\boldsymbol{\beta}_j^b \in \mathbb{R}^{b\times c}$	coefficients of fixed effects for SNPs $j, \ldots, j+b-1$
$\boldsymbol{RSS}_j^b \in \mathbb{R}^b$	residual sums of squares for SNPs $j, \ldots, j+b-1$
$\boldsymbol{RSS}_0^b \in \mathbb{R}^b$	vector containing b copies of the residual sums of squares of the null model
$\boldsymbol{t}_j^b \in \mathbb{R}^b$	test statistics of SNPs $j, \ldots, j+b-1$
${}^{(k)}\sigma_g^2$	genetic variance component of kth permutation
${}^{(k)}\sigma_e^2$	residual variance component of kth permutation
${}^{(k)}\boldsymbol{y} \in \mathbb{R}^n$	k^{th} permutation of phenotype vector y

${}^{(k)}\boldsymbol{V} \in \mathbb{R}^{n\times n}$	variance-covariance matrix ${}^{(k)}\sigma_g^2\boldsymbol{K} + {}^{(k)}\sigma_e^2\boldsymbol{I}$ of kth permutation
${}^{(k)}\boldsymbol{C} \in \mathbb{R}^{n\times n}$	lower triangle matrix with ${}^{(k)}\boldsymbol{C}\,{}^{(k)}\boldsymbol{C}^T = {}^{(k)}\boldsymbol{V}$
${}^{(k)}\boldsymbol{Y}^b \in \mathbb{R}^{b\times n\times 1}$	3D tensor containing b copies of the permutation ${}^{(k)}y$
${}^{(k)}\boldsymbol{C}^b \in \mathbb{R}^{b\times n\times n}$	3D tensor containing b copies of ${}^{(k)}\boldsymbol{C}$
${}^{q}\boldsymbol{X}_j^b \in \mathbb{R}^{q\times b\times n\times c}$	4D tensor containing q copies of $\boldsymbol{X}_j^b$
${}^{q}\boldsymbol{Y}^b \in \mathbb{R}^{q\times b\times n\times 1}$	4D tensor containing ${}^{(1)}\boldsymbol{Y}^b, \dots, {}^{(q)}\boldsymbol{Y}^b$
${}^{q}\boldsymbol{C}^b \in \mathbb{R}^{q\times b)sn\times n}$	4D tensor containing ${}^{(1)}\boldsymbol{C}^b, \dots, {}^{(q)}\boldsymbol{C}^b$
${}^{q}\widetilde{\boldsymbol{X}}_j^b \in \mathbb{R}^{q\times b\times n\times c}$	4D tensor containing the transformed data $\left({}^{q}\boldsymbol{C}^b\right)^{-1}\,{}^{q}\boldsymbol{X}_j^b$
${}^{q}\widetilde{\boldsymbol{Y}}^b \in \mathbb{R}^{q\times b\times n\times 1}$	3D tensor containing the transformed data $\left({}^{q}\boldsymbol{C}^b\right)^{-1}\,{}^{q}\boldsymbol{Y}^b$
$\left({}^{q}\boldsymbol{X}_j^b\right)^T \in \mathbb{R}^{q\times b\times c\times n}$	4D tensor containing the transposed 3D tensors $\left(\boldsymbol{X}_j^b\right)^T, \dots, \left(\boldsymbol{X}_{j+b-1}^b\right)^T$
${}^{q}\boldsymbol{\beta}_j^b \in \mathbb{R}^{q\times b\times c}$	coefficients of fixed effects for SNPs $j, \dots, j+b-1$ and q permutations
${}^{q}\boldsymbol{RSS}_j^b \in \mathbb{R}^{q\times b}$	residual sums of squares for SNPs $j, \dots, j+b-1$ and q permutations
${}^{q}\boldsymbol{RSS}_0^b \in \mathbb{R}^{q\times b}$	matrix containing b copies of the residual sums of squares of the null model for each permutation
${}^{q}\boldsymbol{t}_j^b \in \mathbb{R}^{q\times b}$	test statistics of SNPs $j, \dots, j+b-1$ for q permutations

Supplementary Table 2: Count of true positive (TP) and false positive (FP) phenotypes out of 50 simulated phenotypes, and phenotype-wise false discovery rate (FDR) per effect strength for Bonferroni threshold (bonf) and permutation-based threshold (perm).

threshold	effect	measure	normal	4	3	2	1	0.1
bonf	1.0	TP	4	7	6	4	8	10
bonf	1.0	FP	3	7	13	19	22	49
bonf	1.0	FDR	0.43	0.50	0.68	0.83	0.73	0.83
bonf	1.5	TP	40	39	42	40	42	39
bonf	1.5	FP	7	14	19	25	23	49
bonf	1.5	FDR	0.15	0.26	0.31	0.39	0.35	0.56
bonf	2.0	TP	50	49	48	50	50	48
bonf	2.0	FP	17	23	25	32	25	49
bonf	2.0	FDR	0.25	0.32	0.34	0.39	0.33	0.51
perm	1.0	TP	5	10	7	3	2	1
perm	1.0	FP	7	5	5	5	3	13
perm	1.0	FDR	0.58	0.33	0.42	0.62	0.60	0.93
perm	1.5	TP	46	39	39	31	30	4
perm	1.5	FP	15	15	11	13	6	14
perm	1.5	FDR	0.25	0.28	0.22	0.29	0.17	0.78
perm	2.0	TP	50	49	48	50	48	26
perm	2.0	FP	20	16	17	19	12	18
perm	2.0	FDR	0.29	0.25	0.26	0.28	0.20	0.41

2 Supplementary Figures

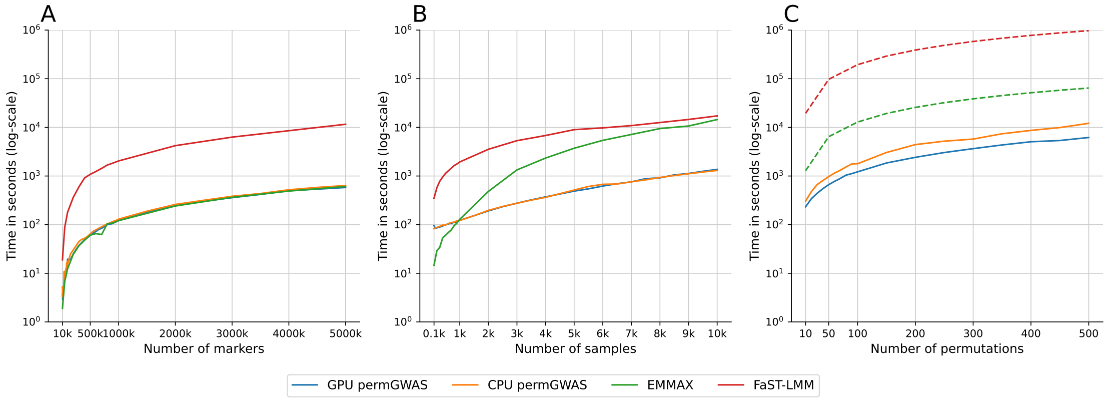

Supplementary Figure 1: Runtime comparison of `permGWAS` vs. `EMMAX` and `FaST-LMM` using 8 cores. Note that all axes are log-scaled. (A) Computational time as function of number of SNPs with fixed number of 1 000 samples. (B) Computational time as function of number of samples with 10^6 markers each. (C) Computational time as function of number of permutations with 1 000 samples and 10^6 markers each. Dashed lines for `EMMAX` and `FaST-LMM` are estimated based on the computational time for 1 000 samples and 10^6 markers times the number of permutations.

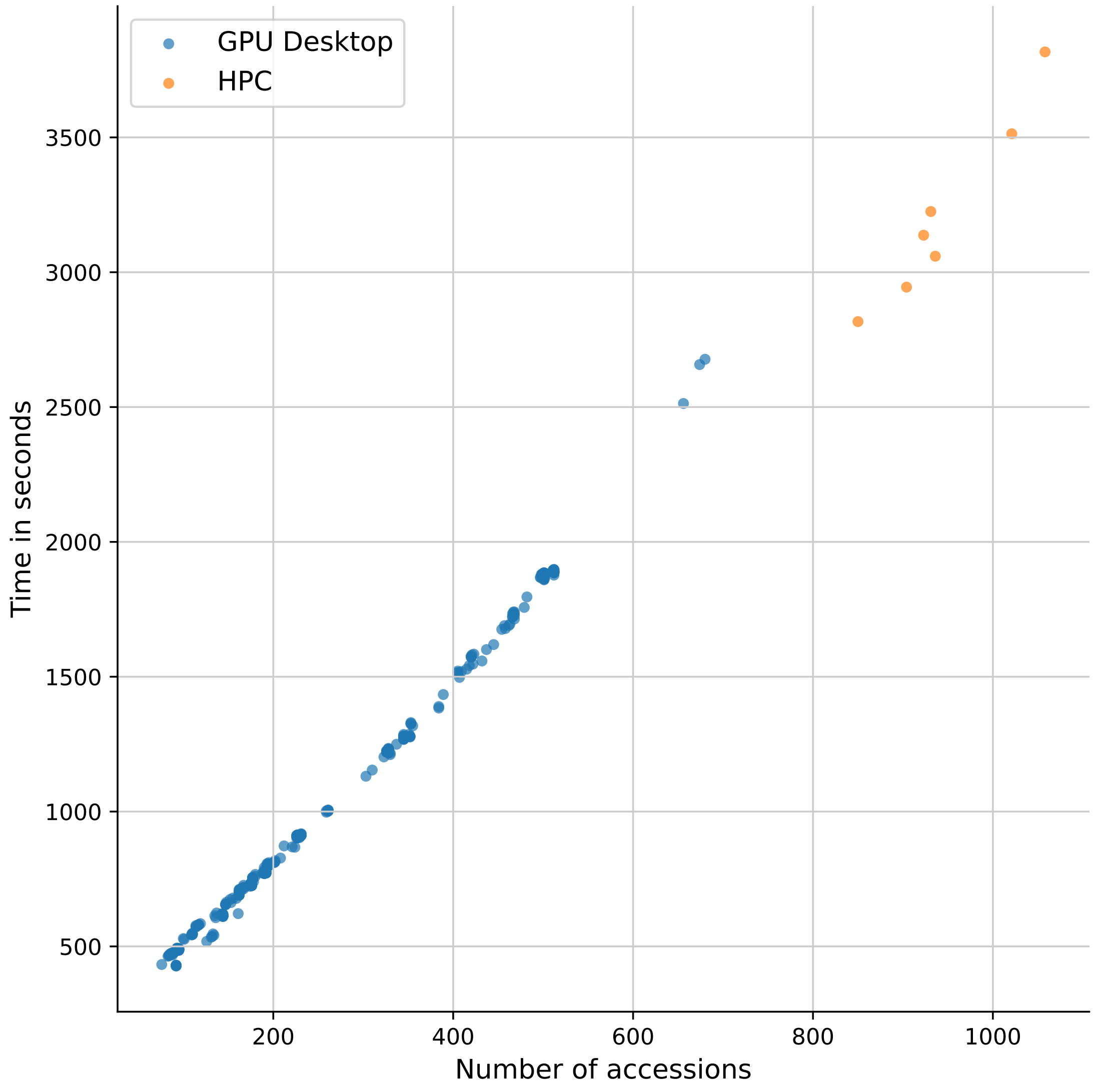

Supplementary Figure 2: Runtime comparison of 516 phenotypes from *Arabidopsis thaliana* using 100 permutations each. Blue dots represent runtime of GWAS on a desktop machine with one Intel Xeon 8 core CPU with 3.5 GHZ, 128 GB of memory and a single NVIDIA RTX A5000 GPU with 24 GB memory. Orange dots are runtime measurements of GWAS on a High Performance Cluster (HPC) including a NVIDIA A100 GPU.

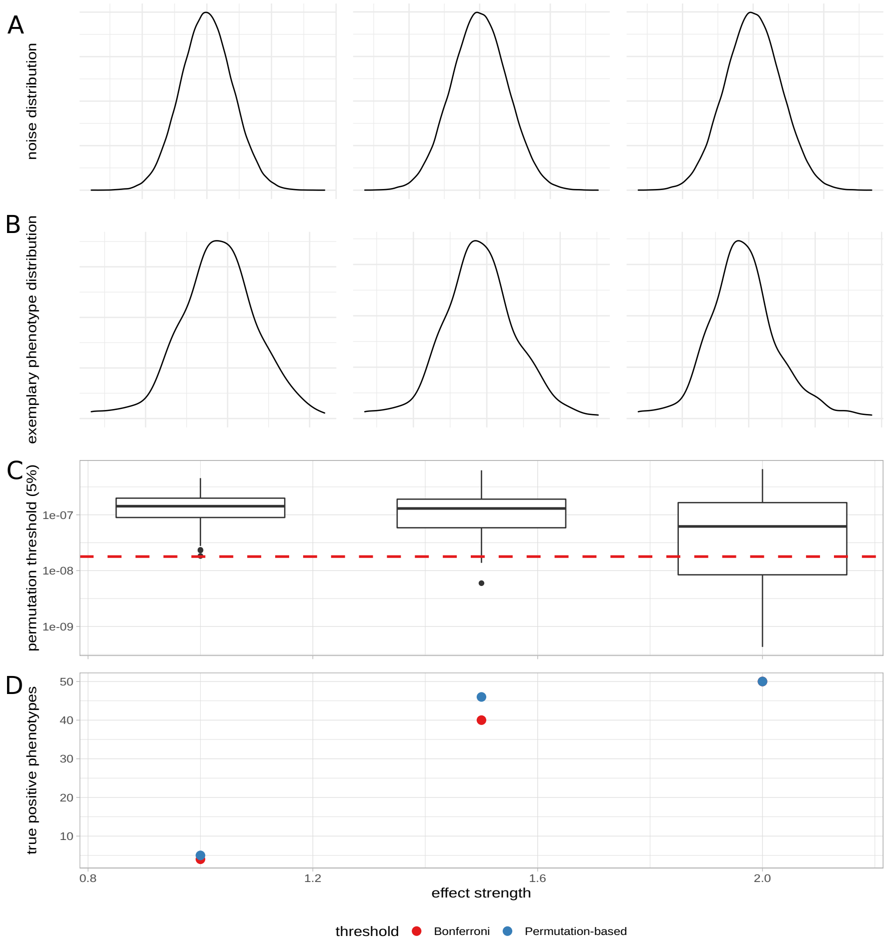

Supplementary Figure 3: Simulated phenotypes with normally distributed noise and different effect strengths. In the simulation the effect strength of the causative SNP was chosen to explain about 10% of total variance. The calculated phenotypic value was then multiplied by a factor (1.0, 1.5, or 2.0) to get different effect strengths. (A) Shape of the noise (normal). (B) Exemplary phenotypic value distribution for each shape parameter. (C) Permutation-based thresholds over 50 simulated phenotypes as box plots for each effect strength. Red dashed line illustrates the fixed Bonferroni significance threshold. (D) Number of true positive (TP) phenotypes (out of 50) for both the fixed Bonferroni significance threshold and the permutation-based significance threshold.

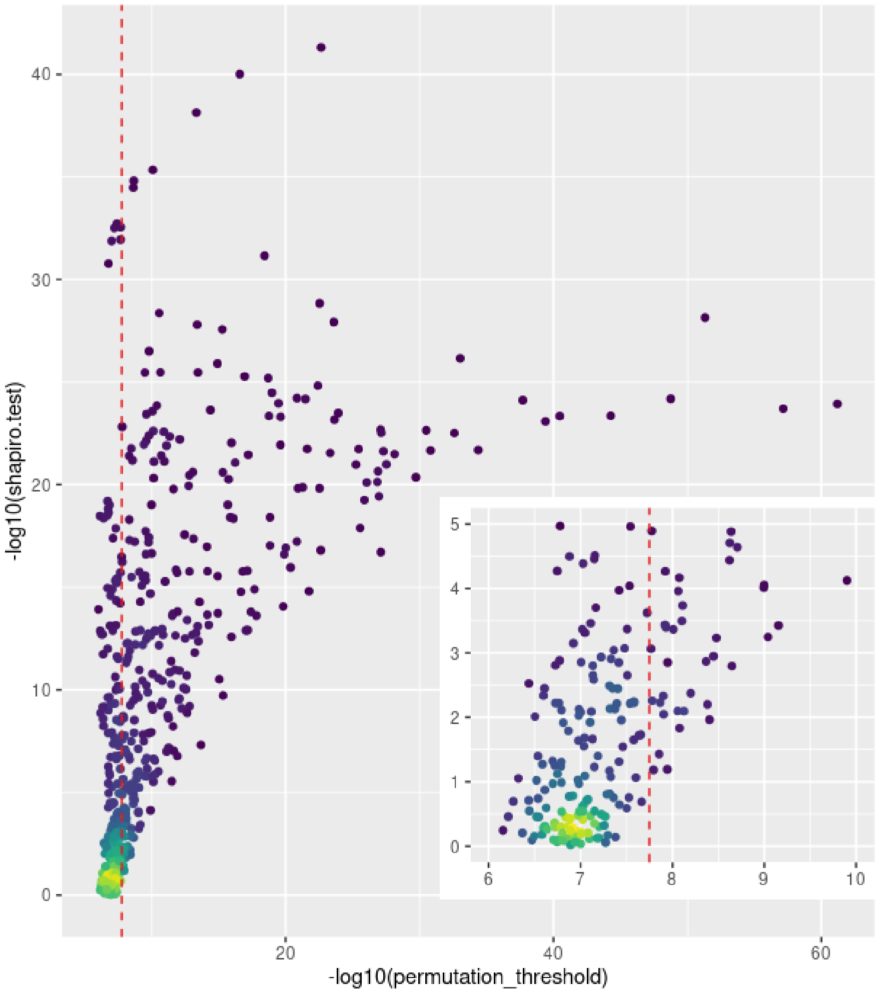

Supplementary Figure 4: Correlation between permutation-based thresholds and the p-value from a Shapiro-Wilk test on the phenotypic distribution of 516 *Arabidopsis thaliana* phenotypes. The static Bonferroni threshold for 2.8 M markers is shown by a red vertical dashed line. Note that the shown threshold is calculated for 2.8 M markers and might differ slightly for phenotypes with small samples sizes. Each dot represents one phenotype and the false colors denote the amount of phenotypes at the same coordinates. The inset enlarges the region for normal and nearly normal distributed phenotypes.

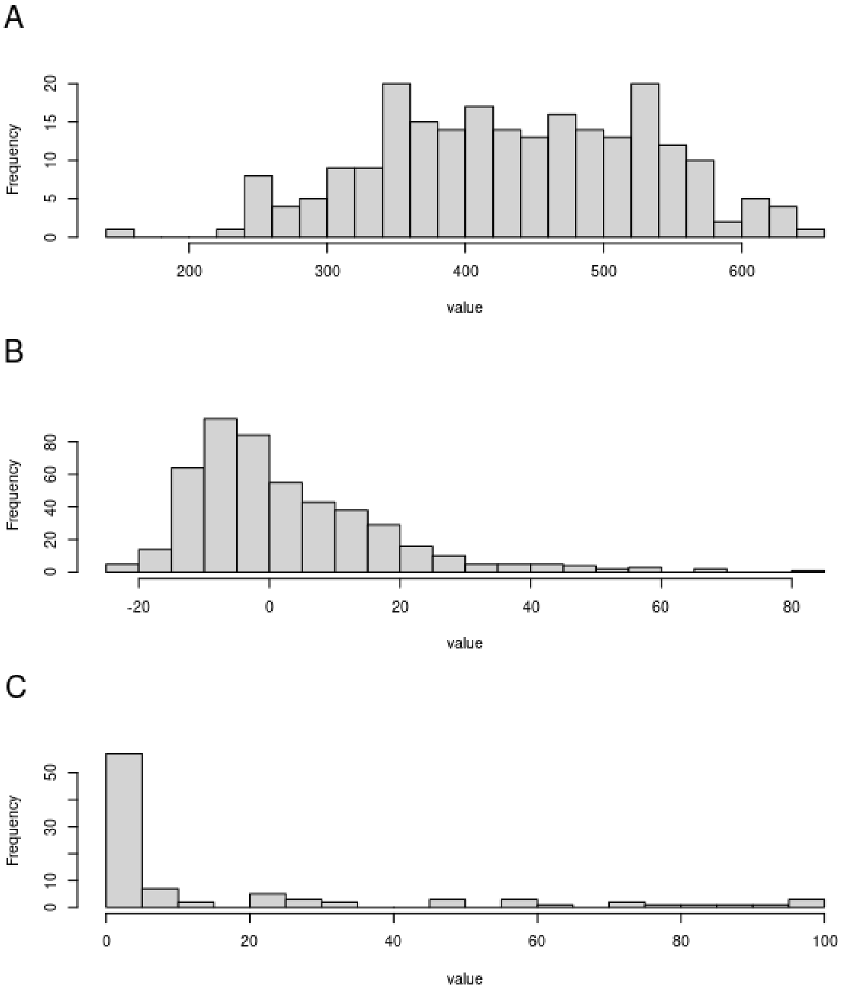

Supplementary Figure 5: Histogram of the phenotypic distribution of three different *Arabidopsis thaliana* phenotypes. (A) Phenotype 744 (https://arapheno.1001genomes.org/phenotype/744/), which is nearly normal distributed (p=0.04). ,(B) Phenotype 118 (https://arapheno.1001genomes.org/phenotype/118/), which is skewed and non-normally distributed (p<1e-17). (C) Phenotype 325 (https://arapheno.1001genomes.org/phenotype/325/), which is zero inflated and also non-normally distributed (p<1e-12).

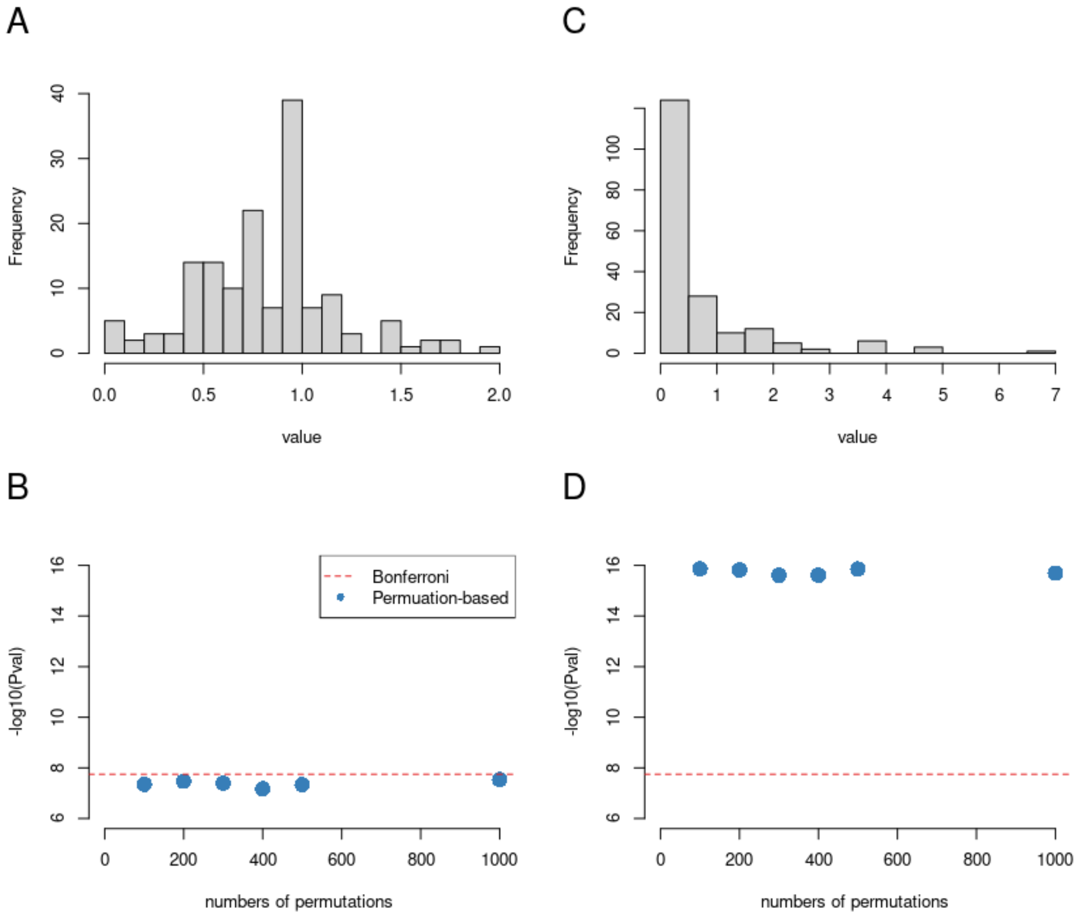

Supplementary Figure 6: Permutation-based thresholds with different amounts of permutations. Two different *Arabidopsis thaliana* phenotypes have been analyzed with varying amounts of permutations. Phenotype 1271 (https://arapheno.1001genomes.org/phenotype/1271/) is nearly normal distributed and shown in (A) and (B) , while phenotype 372 (https://arapheno.1001genomes.org/phenotype/372/) is non-normally distributed ((C) and (D)). The Bonferroni threshold is denoted by a red horizontal dashed line and the respective permutation-based thresholds for different numbers of permutations are shown by blue dots. Note that the latter is stable between 100 and 1000 permutations both for the normal and non-normal distributed phenotype.

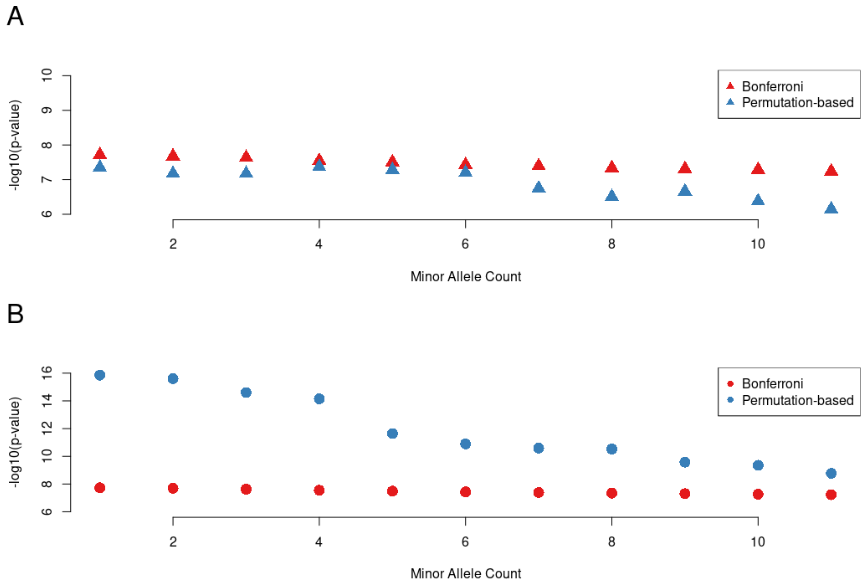

Supplementary Figure 7: The effect of minor allele filters on Bonferroni and permutation-based thresholds. The respective thresholds are shown as a function of increasing minor allele filters, where rare alleles have been removed prior to the analysis. (A) shows a nearly normally distributed phenotype (1271) and (B) a non-normally distributed phenotype (372). The Bonferroni threshold (red) becomes slightly higher with an increasing minor allele filter, as fewer markers are tested. This increase is more pronounced for the permutation-based threshold, especially for the non-normally distributed phenotype. Here the increase in the threshold is non linear, but specific for the phenotypic distribution.

3 Supplementary Files

Supplementary File 1: File includes permutation-based thresholds and number of hits for all 516 AraPheno phenotypes. Supplementary files can be found at: `https://github.com/grimmlab/permGWAS/tree/main/suppl_data`.
